# Invariant NKT cells metabolically adapt to the acute myeloid leukaemia environment

**DOI:** 10.1007/s00262-022-03268-4

**Published:** 2022-08-13

**Authors:** Victoria Stavrou, Livingstone Fultang, Sarah Booth, Daniele De Simone, Arekdiusz Bartnik, Ugo Scarpa, Luciana Gneo, Silvia Panetti, Sandeep Potluri, Meaad Almowaled, Jonathan Barlow, Andris Jankevics, Gavin Lloyd, Andrew Southam, David A. Priestman, Paul Cheng, Warwick Dunn, Frances Platt, Hitoshi Endou, Charles Craddock, Karen Keeshan, Francis Mussai, Carmela De Santo

**Affiliations:** 1 Institute of Immunology and Immunotherapy, University of Birmingham, Birmingham, B15 2TT UK; 2 Institute of Cancer and Genomics, University of Birmingham, Birmingham, B15 2TT UK; 3 Paul O’Gorman Leukaemia Research Centre, University of Glasgow, Glasgow, G12 0YN UK; 4 School of Sport, Exercise and Rehabilitation Sciences, University of Birmingham, Birmingham, B15 2TT UK; 5 School of Biosciences and Phenome Centre Birmingham, University of Birmingham, Birmingham, B15 2TT UK; 6 Department of Pharmacology, University of Oxford, Oxford, OX1 3QT UK; 7 Bio-Cancer Treatment International, Hong Kong Science Park, Hong Kong, China; 8 Institute of Metabolism and Systems Research, University of Birmingham, Birmingham, B15 2TT UK; 9 J-Pharma Co. Ltd, Yokohama, Kanagawa 230-0046 Japan

**Keywords:** iNKT, AML, ASS, LAT-1, Arginine, Cancer

## Abstract

Acute myeloid leukaemia (AML) creates an immunosuppressive environment to conventional T cells through Arginase 2 (ARG2)-induced arginine depletion. We identify that AML blasts release the acute phase protein serum amyloid A (SAA), which acts in an autocrine manner to upregulate ARG2 expression and activity, and promote AML blast viability. Following in vitro cross-talk invariant natural killer T (iNKT) cells become activated, upregulate mitochondrial capacity, and release IFN-γ. iNKT retain their ability to proliferate and be activated despite the low arginine AML environment, due to the upregulation of Large Neutral Amino Acid Transporter-1 (LAT-1) and Argininosuccinate Synthetase 1 (ASS)-dependent amino acid pathways, resulting in AML cell death. T cell proliferation is restored in vitro and in vivo. The capacity of iNKT cells to restore antigen-specific T cell immunity was similarly demonstrated against myeloid-derived suppressor cells (MDSCs) in wild-type and Jα18^−/−^ syngeneic lymphoma-bearing models in vivo. Thus, stimulation of iNKT cell activity has the potential as an immunotherapy against AML or as an adjunct to boost antigen-specific T cell immunotherapies in haematological or solid cancers.

**Supplementary Information:**

The online version contains supplementary material available at 10.1007/s00262-022-03268-4.

## Introduction

T cell immunotherapies, notably chimeric-antigen receptor T (CAR-T) cells against CD19 or CD22 on acute lymphoblastic leukaemia (ALL) blasts, have demonstrated the potential to generate clinically significant immune responses in haematological malignancies [1]. However, in AML activating T cell immunity against blasts remains challenging. Strategies to induce T cell cytotoxicity against AML blasts including upregulation of peptide antigen presentation, release of T cells from immune checkpoint inhibition, and engineering of antigen-specific or chimeric-antigen receptors have not consistently demonstrated activity in clinical trials [2–4].

The failure of T cell responses against AML blasts is dependent on the interplay of both T cell factors and blast phenotype. T cells from AML patients are reported to demonstrate increased exhaustion, conversion to a Treg phenotype, and failure to generate an immune synapse that leads to activation of cytotoxicity [5, 6]. Concurrently, blasts evade T cell responses through the downregulation of HLA-dependent antigen presentation, expression of inhibitory cell surface molecules, release of immunosuppressive cytokines, and consumption of amino acids critical to T cell expansion [7, 8]. These immunosuppressive strategies are similarly employed by the non-malignant population MDSCs to impair T cell expansion in the setting of solid and haematological cancers [9–11]. Previously, we identified that arginine catabolism by ARG2 contributes to both AML blast viability and suppression of peptide antigen-specific T cell responses in patients [7, 12–14]. ARG2 immunomodulatory activity has since been described in a number of pathological settings, and however, the factors which regulate the enzyme’s expression are still poorly understood, including in AML. Inflammatory cytokines may drive malignant transformation or expansion in some AML patients at diagnosis [15].

Although T cells represent a major component of the body’s response to cancer, the potential of other immune populations to generate anti-leukaemia immunity or support T cell immunity in solid cancers has not been fully exploited. Here, we investigate how iNKT cells are adapted to the immunosuppression created by AML or MDSCs, resulting in a direct reduction of cancer burden and restoration of T cell function.

## Methods

### Patient samples and study approvals

Blood samples were obtained from 72 AML patients at diagnosis treated at University Hospitals Birmingham and Birmingham Children’s Hospital, UK. An additional cohort of samples (n = 42) was collected from AML patients ineligible for intensive chemotherapy treated with either azacitidine or azacitidine and vorinostat in a multi-centre, randomised phase II trial (RAVVA; NCT01617226) [14]. Fresh peripheral blood mononuclear cells (PBMCs) were separated using a Lymphoprep (StemCell Technologies) gradient and enriched based on CD33 or CD34 expression using anti-human CD33 or anti-human CD34 magnetic beads (Miltenyi) following manufacturer’s instructions. All samples were processed in assays within 12 h of blood sampling. Peripheral blood samples from healthy donors were obtained from the University of Birmingham. In accordance with the Declaration of Helsinki, all samples were obtained after written, informed consent prior to inclusion in the study. Regional Ethics Committee (REC Number 10/H0501/39) approval for the study was granted.

### Flow cytometric analysis

Whole blood and PBMCs were stained with human CD1d PBS-57 Tetramer (NIH Tetramer Centre, Emory USA) at 37 °C for 20 min. Following a wash in FACS Buffer, cells were stained for surface antigens: anti-human CD3 (clone UCHT1/HIT3a), CD33 (clone HIM3.4/WM5.3), CD34 (clone 581), FPR2 (clone K102B9), TLR2 (clone TL2.2), TLR4 (clone HTAR5), CD1d (clone 541), CD40 (clone 5C3), CD40L (clone 24–31), FAS (clone DX2), CD69 (clone FN50), CD38 (clone HB-7) antibodies (BioLegend), TCR Vα24 (clone REA948), TCR Vβ11 (clone REA 559) (Miltenyi Biotec) on ice for 30 min, where indicated. For murine analysis, cells were stained with murine CD1d PBS-57 tetramer (NIH Tetramer Service, USA), SIINFEKL tetramer (Biolegend), and anti-murine CD11b (clone M1/70), GR1 (clone 1A8), CD45.1 (clone A20), CD45.2 (clone 104), CD1d (clone 1B1) and CD3 (clone 17A2) (eBiosciences) as indicated. Propidium iodide (PI) (Biolegend) was used to assess viability. Intracellular staining for IFN-γ (clone 4S.B3; BioLegend), CD107a (clone H4A3; Biolegend), or ASS1 (Abcam), and goat anti-rabbit IgG (isotype control;Abcam) proteins was determined according to manufacturer’s instructions (BioLegend). Where indicated CFSE (Thermo Fisher Scientific) was used to assess AML or iNKT proliferation. CFSE (1:1000 dilution) was added to target cells for 20 min at 37 °C. The assay was quenched with complete media for 5 min, before washing and resuspending. Cells were analysed using a Beckman Coulter Cytoflex flow cytometer and analysed using FlowJo and CytExpert software (Tree Star Inc).

### Murine experiments

Generation of the immortalised MLL-AF9 murine AML cell line has been previously described [14, 16, 17]. MLL-AF9 cells were thawed and resuspended and 0.5 × 10^6^ MLL-AF9 cells were transplanted intravenously into sublethally irradiated (4.5 Gy) B6.SJL-Ptprc^a^ Pepc^b^/BoyJ (CD45.1 +) mice recipients. αGalCer (2 μg/mouse) or vehicle was injected intravenously as indicated in the individual experiments, before the mice were sacrificed at day 17 post-bone marrow transplant. Serum was collected by tail vein sampling on Day 12. AML donor cells (CD45.2 +), iNKT and T cell frequency were identified by flow cytometry of the blood, bone marrow flushed from the legs, and mechanically disrupted spleens following red cell lysis. All vehicle controls were treated with PBS iv.

EG7 lymphoma cells (5 × 10^5^) were engrafted subcutaneously into C57BL/6 and iNKT cell-deficient mice (Jα18 TCR gene segment, TCRa J^+m1Tgi^, Jα18^−/−^). Mice were injected with 1 μg αGalCer /mouse following ovalbumin (OVA) vaccine injection (10^6^ PFU/mouse) as described previously [18]. Tumour progression, MDSC and OVA-specific T cell expansion were measured. Splenocytes from OT-1 T cell receptor (TCR) transgenic mice (5 × 10^6^) were injected into C57BL/6 and Jα18^−/−^ mice, followed by OVA-peptide (20 ng/ml) pulsed dendritic cells (2 × 10^6^). MDSCs were previously isolated by MACS sorting (MDSC Isolation Kit, Miltenyi Biotec) from EG7 tumours engrafted in mice as described above. 2 × 10^6^ MDSCs were injected iv followed by αGalCer (2 μg /mouse) where indicated.

Wild-type C57BL/6 mice were treated with 10 mg/kg recombinant human arginase (BCT-100, BCTI, Hong Kong) or PBS iv daily. On day 2, mice were treated with αGalCer (2 μg /mouse) or vehicle iv and all mice were sacrificed on day 5. Blood, bone marrow, and spleens were analysed by flow cytometry as before. Procedures were carried out in accordance with UK Home Office Guidelines.

### Expansion of human iNKT cells

Human iNKT cells were isolated as previously described [18]. In short, PBMCs were isolated from healthy donors' buffy coats by density gradient centrifugation over Lymphoprep™ (StemCell Technologies). PBMCs were cultured in complete RPMI, supplemented with β-mercaptoethanol (1X) (SIGMA), in the presence of αGalactosylceramide (αGalCer, 100 ng/ml) (BioVision) for 14 days. After 3 days 1000U/ml IL-2 (Novartis) was added to cultures. iNKT cells were isolated by sorting PBMCs labelled with CD1d tetramer (NIH Tetramer service, USA) using a BD FACS Aria Fusion cell sorter. Thereafter, cells were co-cultured with allogenic irradiated PBMCs stimulated with PHA (1 µg/ml) (Gibco) and fed every 3–4 days with fresh medium containing 1000 U/ml IL-2 to create ‘iNKT cell lines’. iNKT cell frequncy was determined using CD1d tetramer (NIH Tetramer Service, Emory USA), Vα24 antibody and Vβ11 (Miltenyi Biotec). Where indicated primary human iNKT cells were isolated fresh by flow sorting, using human CD1d PBS-57 Tetramer (NIH Tetramer Centre, Emory USA).

### iNKT cell rescue of T cell proliferation assay

T cells were prepared from healthy donors as described above. 2 × 10^5^ T cells, enriched by negative selection (Miltenyi Biotec), were cultured with allogenic irradiated (5000 rad) dendritic cells (DC, 0.5 × 10^5^), in 200 RPMI 5% human serum (Sigma) in 96 well flat bottom plates. Cells were incubated at 37^0^C, 5% CO_2_ for 4 days. 1 mCi/ well ^3^H-thymidine (PerkinElmer Life Sciences) was added for 12–16 h. ^3^H-thymidine incorporation was measured using a Wallac Microbeta Jet 1450 reader (PerkinElmer). AML inhibition of T cell proliferation was carried out by co-culturing AML blasts from patients with healthy donor T cells and irradiated DCs. To test the ability of iNKT cells to overcome AML-induced suppression of T cell proliferation 0.25 × 10^5^ iNKT cells were first co-cultured with AML blasts (1 × 10^5^) for 8 h, in the presence of αGalCer (100 ng/ml). After 8 h, the cells were washed and T cells and DC were added to cultures according to the protocol above. Data are expressed as a percentage of T cell proliferation driven by allogenic irradiated DCs in the presence of AML cells, as compared to allogenic PBMC proliferation in the absence of AML cells (100%). Where indicated iNKT cells were cultured with anti-CD3 (3 μg/ml)/anti-CD28 (2 μg /ml) in the presence of JPH203 (0.125 mM; J-Pharma, Japan). DMSO was used as a vehicle control.

### iNKT-AML cytotoxicity assay

AML patient blasts and AML cell lines were cultured with iNKT cells and αGalCer (100 ng/ml) for 24 h, 48 h, and 72 h. Supernatants were harvested to determine cytokine release, and viability of AML blasts was determined by flow cytometry, using propidium iodide (BD Pharmingen) staining.

### Statistics

A Mann–Whitney t-test was used to determine the statistical significance of the difference in unpaired observations between 2 groups (GraphPad Prism, USA). A Wilcoxon matched paired t test was used to determine the statistical significance of the difference in paired observations between 2 groups (GraphPad Prism, USA). *p* values are two-tailed and where values were < 0.05,they were considered statistically significant. For bioenergetic analyses, statistical differences were assessed using one-way ANOVA with Fisher’s LSD post hoc test.

## Results

### Serum amyloid A1 (SAA1) upregulates ARG2 in AML blasts

**Fig. 1:**
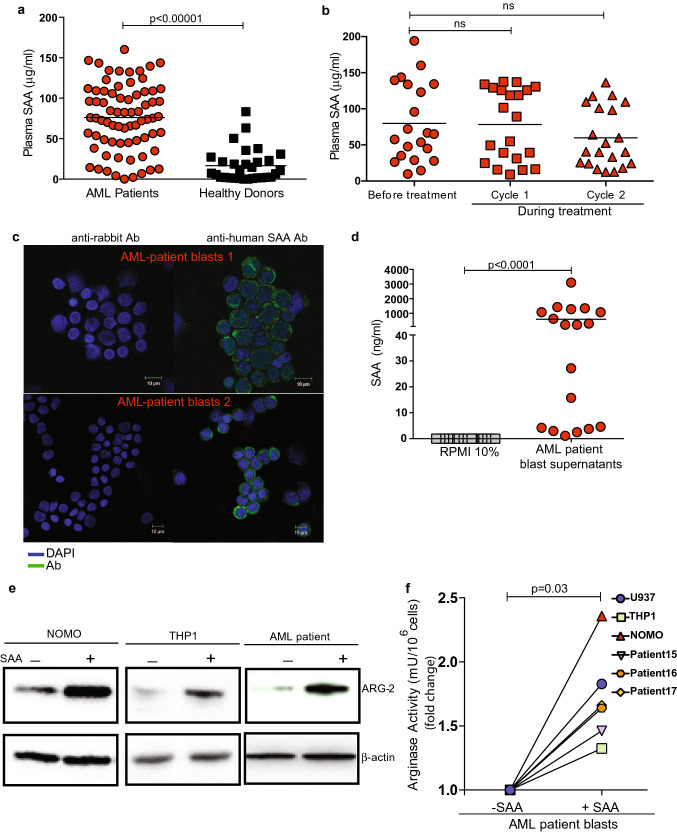
SAA upregulates ARG2 in AML blasts. **a** ELISA for SAA in the plasma from n = 72 newly diagnosed AML patients prior to treatment, compared to levels in n = 27 healthy donors. *P* value determined by unpaired t-test. **b** Plasma SAA concentrations remain unchanged in n = 20 AML patients treated with cycles of azacitidine/vorinostat as measured by ELISA. *P* value determined by paired t-test. **c** Confocal microscopy of AML blasts from patients showing intracellular expression of SAA. DAPI—blue, SAA—green, (n = 2 individual donors). **d** Increased SAA in the supernatants of AML blasts cultured for 48 h in R10%, measured by ELISA (n = 18 patients). Each dot is the mean of duplicates. *P* value determined by unpaired t-test. **e** SAA (10 μg/ml) leads to upregulation of ARG2 in AML cells, measured by western blot at 72 h. Actin is shown as a loading control. Representative of n = 3 individual experiments. **f** Increased arginase activity in AML patients’ blasts and cell lines following SAA (10 μg /ml) treatment for 72 h, as measured by the conversion of arginine into urea. Each dot is the mean of duplicate samples. *P* value determined by paired t-test

AML blasts induce a low arginine environment in which the immune system must function (Supp Fig. 1a, b). We have previously shown that inflammatory mediators can modulate the phenotype of immunosuppressive myeloid cells, and hypothesised such factors could control ARG2 expression in AML blasts [19]. Evaluation of the plasma of AML patients at diagnosis identified no significant differences in the concentrations of Th1/ Th2 cytokines (Supp Fig. 1c, d). However, SAA levels were significantly raised in AML patients compared to healthy controls (Fig. 1a), and persistent during treatment in a second cohort of patients (Fig. 1b) [14]. Confocal microscopy revealed blasts express SAA (Fig. 1c), which can be released (Fig. 1d) [20, 21].

AML blasts were treated with recombinant SAA, leading to an increase in blast viability ex vivo (Supp Fig. e, f). Evaluation of cell lines and patients’ blasts identified expression of the SAA receptors Toll-like Receptors: TLR2, TLR4, and Formyl-Peptide Receptor 2 (FPR2) (Supp Fig. 2a). Sequential blockade reveals SAA signals through FPR2 (Supp Fig. 2b, c), contributing to local inflammation by increasing the production and release of IL-1β (Supp Fig. 2d, e). Notably, SAA leads to the upregulation of intracellular ARG2 expression and activity (Fig. 1e, f), and a corresponding reduction in extracellular arginine (Supp Fig. 2f).

### iNKT cells are adapted to the low arginine microenvironment through LAT-1 and ASS1 expression

**Fig. 2:**
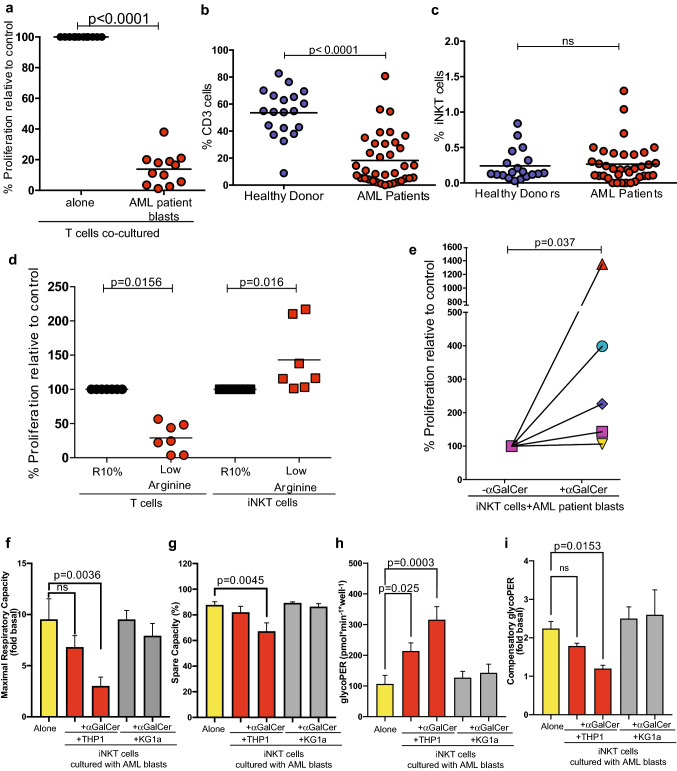
iNKT cells are activated following cross-talk with AML blasts. **a** AML blasts suppress T cell proliferation in a mixed leukocyte reaction assay (allogeneic T cells and dendritic cells co-cultured for 96 h). n = 12 AML patients. Each dot is the mean of duplicates. *P* value determined by paired t-test. **b** The percentage of CD3 + T cells in the blood of healthy donors (n = 19) and AML patients (n = 37) at diagnosis measured by flow cytometry. *P* value determined by unpaired t-test. **c** The percentage of iNKT cells in the blood of healthy donors (n = 19) and AML patients (n = 37) at diagnosis, measured by flow cytometry with CD1d tetramer staining. *P* value determined by unpaired t-test. **d** Anti-CD3/anti-CD28 antibody-driven iNKT cell line proliferation (n = 7 donors) is unaffected by in vitro culture in low arginine conditions as determined by flow cytometry, after 96 h. Proliferation of T cells (n = 7donors) is suppressed in low arginine. Each dot represents the mean of duplicate samples. *P* value determined by paired t-test. **e **αGalCer (100 ng/ml) presentation by AML blasts induces iNKT cell line proliferation, as measured by flow cytometry after 72 h. Each dot represents the mean of duplicate samples. *P* value determined by paired t-test. f) Mitochondrial oxygen consumption rates (OCR) in the absence and presence of oligomycin were assessed in iNKT cells ± THP1 or KG1a ± αGalCer (100 ng/ml pulsed for 4 h) to establish maximal respiratory capacity of iNKT cell line, expressed as fold change between BAM 15-induced OCR and baseline OCR. **g** Spare respiratory capacity of iNKT cell lines was calculated as the difference between BAM 15-induced OCR and baseline OCR, expressed as percentage of maximal respiration. Glycolytic proton efflux rates (glycoPER) in the absence and presence of oligomycin were probed in iNKT cell lines to establish baseline rates of glycolysis **h** and compensatory glycolysis (i), respectively. Mitochondrial OCR were corrected for non-mitochondrial respiration by subtracting OCR following rotenone and antimycin addition. GlycoPER were corrected for non-glycolytic acidification by subtracting remaining PER following 2-DG addition. Data are means ± SEM from 4 cell donors assessed from two independent microplates each containing 3–4 well replicates per donor. Statistical differences were assessed using one-way ANOVA with Fisher’s LSD post hoc test

The failure of immune surveillance contributes to leukaemia progression. Although we and others have shown that T cells numbers are suppressed in the low arginine AML niche ex vivo (Fig. 2a), and in patients (Fig. 2b), we observe that the specialised population of invariant natural killer T cells (iNKT) remains unchanged (Fig. 2c, Supp Fig. 3a) [7]. To investigate the capacity of iNKT to respond in low arginine conditions, sorted cells were stimulated using anti-CD3/anti-CD28 antibodies (Supp Fig. 3b). Although iNKT cells proliferated under low arginine, conventional T cell proliferation was suppressed (Fig. 2d).

iNKT cells recognise glycolipid antigens presented by myeloid cells via the MHC-like molecule CD1d in combination with CD40 co-stimulation. Flow cytometry identified CD1d and CD40 are expressed on patients’ blasts (Supp Fig. 3c, d). Next, we investigated antigen-specific iNKT proliferation by AML under stimulation with αGalCer, a synthetic glycolipid that is an established activator of iNKT cells used in experimental models and clinical trials [22]. Antigen-induced iNKT proliferation was unaffected by AML conditions (Fig. 2e, Supp Fig. 3e). Oxidative and glycolytic flux of iNKT cells was probed using an Agilent Seahorse XFe96 Analyzer. Despite modest differences, neither basal mitochondrial nor ADP phosphorylation-linked respiration were significantly altered between iNKT cells and those co-cultured with AML ± αGalCer (Supp Fig. 3f–i). However, crosstalk of iNKT cells with AML significantly lowered maximal mitochondrial respiratory capacity of iNKT cells from 9.5-fold of basal to threefold of basal when treated with αGalCer (Fig. 2f). Spare respiratory capacity was also significantly lower (Fig. 2g). No significant differences were observed on either maximal respiratory capacity or spare respiratory capacity in iNKT cells co-cultured with the CD1d negative line KG1a ± αGalCer (Fig. 2f, g). Glycolytic Proton Efflux Rate (GlycoPER) was significantly increased in iNKT cells after AML crosstalk (Fig. 2h), and oligomycin-induced compensatory glycoPER is lower in iNKT cells after crosstalk with THP1 but not KG1a ± αGalCer (Fig. 2i). Consistent with this, rates of ATP synthesis linked to glycolysis were significantly increased in iNKT cells after THP1 co-culture ± αGalCer when compared to iNKT cells alone or those co-cultured with KG1a ± αGalCer (Supp Fig. 3j). These data suggest a dependence on glycolytic flux to meet cellular ATP demand in the early phase of activation in iNKT cells. Notably, the lower spare respiratory capacity and increased glycolytic rates of ATP synthesis observed after iNKT cells are cultured with CD1d + AML are consistent with the metabolic changes that occur upon naïve T cell activation [23, 24].

**Fig. 3:**
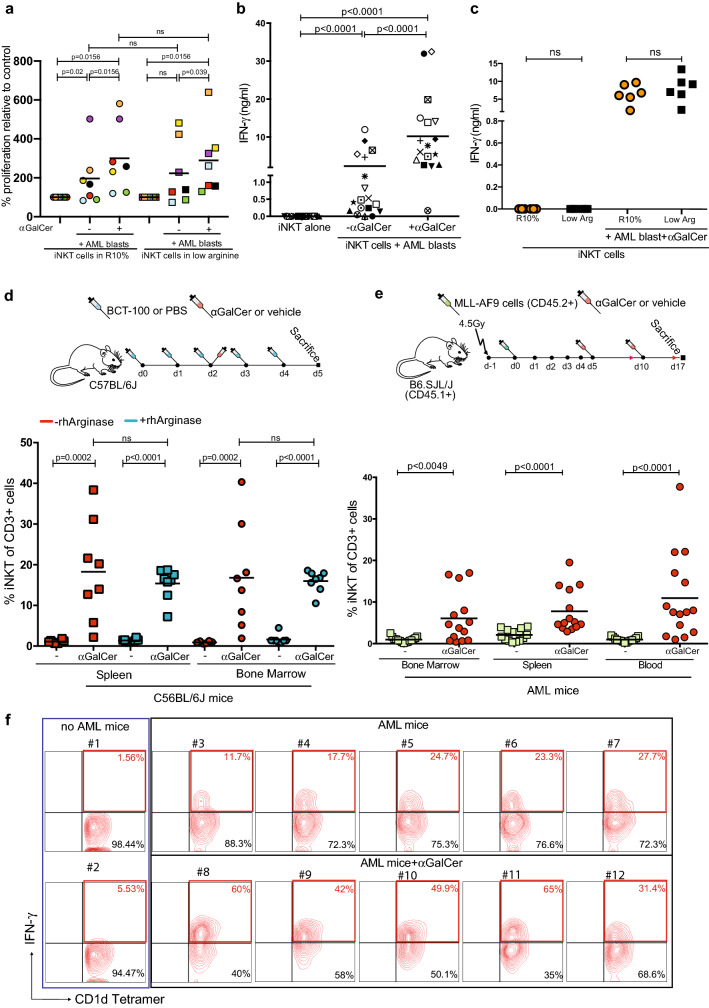
iNKT cells remain activated within the low arginine AML environment in vitro and in vivo. **a **αGalCer (100 ng/ml) presentation by AML blasts induces iNKT cell line proliferation, which is unaffected by low arginine media conditions as measured by flow cytometry after 72 h (blasts from n = 7 AML patients). Each dot represents the mean of duplicate samples. *P* value determined by paired t-test. **b** iNKT cell lines cultured with AML blasts for 72 h (n = 17 AML patients) in the presence or absence of 100 ng/ml αGalCer, release IFN-γ into culture supernatants as measured by ELISA. Each dot represents the mean of duplicates. *P* value determined by paired t-test. **c **αGalCer (100 ng/ml) presentation by AML blasts (n = 6 AML patients) induces iNKT cell line IFN-γ release in vitro after 72 h, which is unaffected by low arginine conditions as measured by ELISA. Representative data of duplicate experiments. *P* value determined by paired t-test. **d** Schematic depicting experimental design. C57BL/6J mice were treated with PBS or recombinant Arginase (BCT-100, 10 mg/kg) in combination with vehicle or 2 μg /mouse αGalCer as indicated. iNKT cell expansion secondary to αGalCer treatment (iv) is unaffected in C57BL/6J mice (n = 8) treated with BCT-100 recombinant human Arginase (rhArginase) (iv) as determined by flow cytometry. Representative of two individual experiments. *P* value determined by paired t-test. **e** Schematic depicting experimental design. CD45.1 + B6.SJL/J mice were irradiated (4.5 Gy) and engrafted with murine MLL-AF9 AML blasts (CD45.2). Following engraftment mice were treated with vehicle or 2 μg /mouse αGalCer as indicated. iNKT cells (CD1d tetramer positive) proliferate following treatment of MLL-AF9 leukaemia bearing mice (n = 15) with αGalCer as measured by flow cytometry. Data from n = 3 individual experiments. *P* value determined by unpaired t-test. **f** Flow cytometry plots of iNKT cells from MLL-AF9 leukaemia bearing mice (n = 14) treated with 2 μg /mouse αGalCer showing upregulation of IFN-γ production as measured by intracellular staining. iNKT cells are detected by CD1d tetramer staining. Control mice with no AML or αGalCer are shown for comparison. Representative data from two individual experiments

Functional evidence for the above activation findings was confirmed. Stimulation of iNKT proliferation by AML with αGalCer was unaffected, even under lower arginine conditions in vitro (Fig. 3a). Culture of iNKT with AML blasts leads to activation-induced IFN-γ production and release (Supp Figs. 4a, 3b), which is also unaffected by low arginine media conditions in vitro (Fig. 3c). Increased levels of the activation markers CD69, CD38 and FAS are similarly observed in both conditions (Supp Fig. 4b, c).

To support these findings immunocompetent, mice were depleted of arginine using recombinant arginase (Supp Fig. 4d). In vivo iNKT cells retained the capacity to expand (Fig. 3d, Supp Fig. 4e) and release IFN-γ (Supp Fig. 4f) on αGalCer antigen stimulation. In an immunocompetent AML murine model (MLL-AF9) that replicated a low arginine environment (Supp Fig. 1b), we similarly demonstrate iNKT cells proliferate in bone marrow, spleen, and blood environments of AML-bearing mice (Fig. 3e) [14, 17]. Activated iNKT within the bone marrow produce and release IFN-γ (Fig. 3f, Sup Fig. 4g, h).

**Fig. 4:**
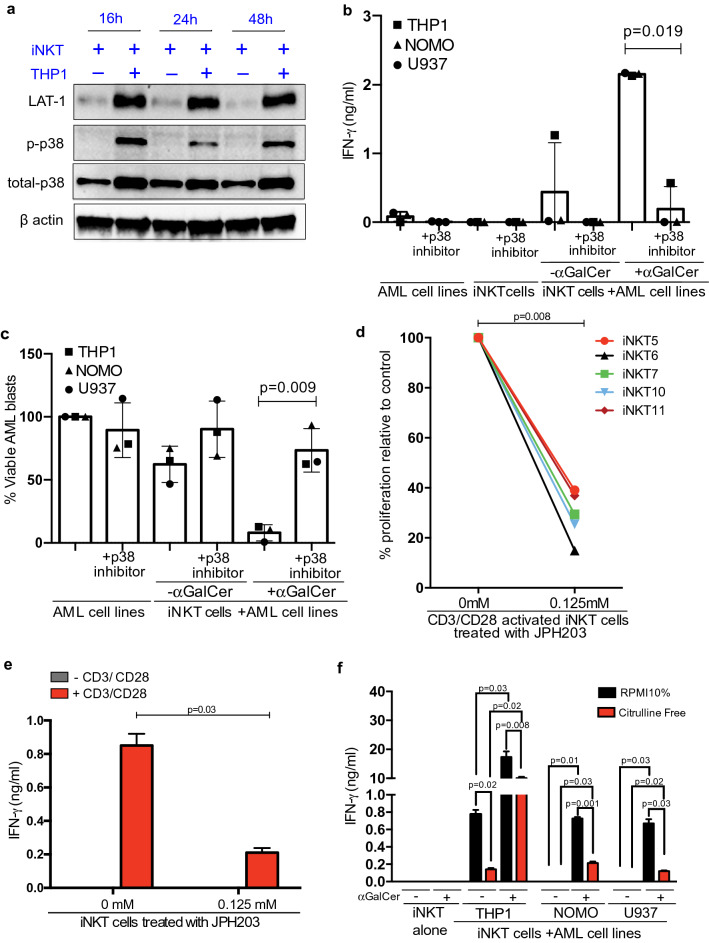
iNKT cell proliferation is dependent on citrulline uptake via LAT-1. **a** LAT-1 and p-p38 expression are increased in sorted iNKT cell lines following incubation with AML in the absence of αGalCer(100 ng/ml) for 72 h. Western blot with actin as a loading control. **b** p38 inhibition (SB203580; 10 μM) in iNKT cell lines inhibits IFN-γ production after cross-talk with AML cell lines. Each dot is the mean of duplicates. *P* value determined by paired t-test. **c** p38 inhibition (SB203580;10 μM) in iNKT cell lines inhibits cytotoxicity against AML blasts. Each dot is the mean of duplicates. *P* value determined by paired t-test. **d** Primary iNKT cell proliferation (n = 5 donors) after 72 h of culture is inhibited by the LAT-1 inhibitor JHP203 (0.125 mM), measured by flow cytometry. Each dot is the mean of duplicates. *P* value determined by paired t-test. **e** LAT-1 inhibition with JPH203 (0.125 mM) impairs primary iNKT activation-induced IFN-γrelease following stimulation with anti-CD3/CD28 antibodies for 72 h, as measured by ELISA. Each dot is the mean of duplicates. *P* value determined by paired t-test. **f** iNKT αGalCer (100 ng/ml) cell line activation-induced IFN-γrelease is inhibited in the absence of citrulline, in co-cultures with AML cell lines for 72 h. *P* value determined by paired t-test

We hypothesised that the iNKT cells may be adapted to low arginine environments by switching away from arginine metabolism. Sorted iNKT cells activated in low arginine conditions were subject to ultra-high performance liquid chromatography mass spectrometry (UHPLC-MS) analysis and demonstrated metabolic perturbations in the arginine and proline metabolism pathway as expected. Pathway enrichment analysis revealed changes in *valine, leucine and isoleucine biosynthesis*, *glycine, serine and threonine metabolism*, *alanine, aspartate and glutamate metabolism*, and *cysteine and methionine metabolism*. (Supplementary Table 1) Consistent with this, isolated iNKT cells post-cross-talk with AML cell lines have upregulation of the transmembrane amino acid transporter LAT-1 (SLC7A5) which mediates uptake of neutral amino acids (Fig. 4a) [25]. LAT1 upregulation is increased with accompanying p38 phosphorylation (Fig. 4a). Inhibition of p38 phosphorylation abrogates iNKT IFN-γ release and cytotoxicity (Fig. 4b, c). Inhibition of LAT-1 with the small molecule inhibitor JPH-203 impairs human iNKT proliferation (Fig. 4d), and IFN-γ release (Fig. 4e), with no effect on viability (Supp Fig. 4i).

**Fig. 5:**
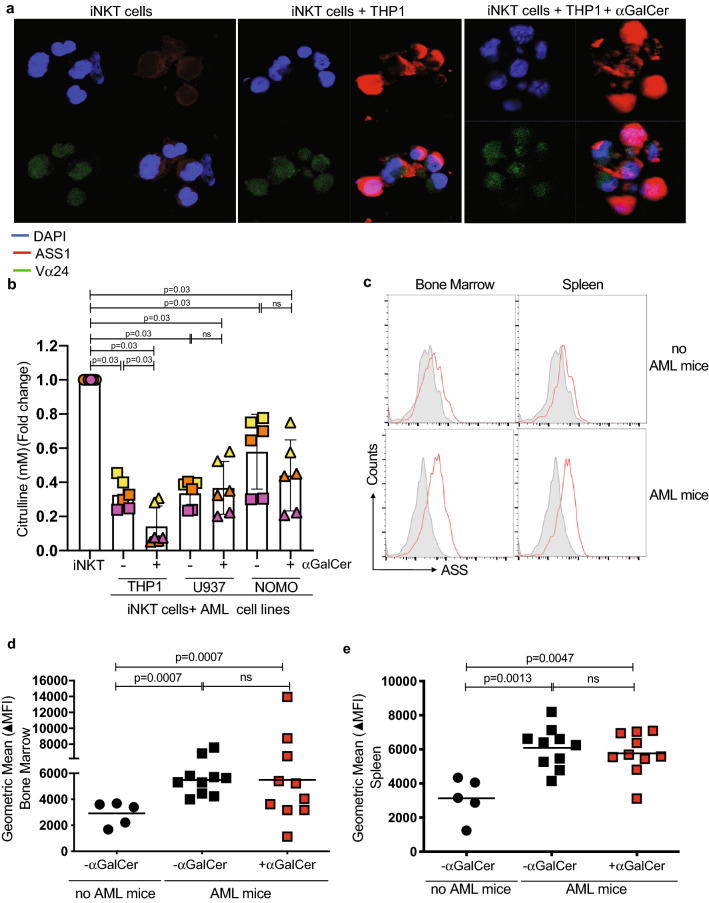
ASS catabolises citrulline in iNKT Cells. **a** Confocal microscopy of primary sorted iNKT cells showing increased ASS1 expression following crosstalk with THP-1 cells for 72 h. DAPI—blue, ASS-1—red, and Vα24—green. **b** iNKT cell lines have increased ASS1 enzyme activity, following cross-talk with AML blasts for 72 h. ASS1 enzyme activity of sorted iNKT cells lysates was determined by citrulline depletion using a colorimetric assay. Data from n = 3 individual experiments. *P* value determined by paired t-test. **c** Representative flow cytometry histograms of iNKT cells sorted from the bone marrow and spleens of n = 10 MLL-AF9 leukaemia bearing mice, demonstrating ASS expression as measured by intracellular staining. Data from n = 2 individual experiments. **d** Geometric means for ASS intracellular staining of iNKT cells sorted from the bone marrow of n = 10 MLL-AF9 leukaemia bearing mice, demonstrating increased ASS expression compared to non-leukaemia control mice in the presence or absence of 2 μg /mouse αGalCer. Data from n = 2 individual experiments. *P* value determined by unpaired t-test. **e** Geometric means for ASS intracellular staining of iNKT cells sorted from the spleens of n = 10 MLL-AF9 leukaemia bearing mice, demonstrating ASS expression compared to non-leukaemia control mice in the presence or absence of 2 μg /mouse αGalCer. Data from n = 2 individual experiments. *P* value determined by unpaired t-test

Complimentary to data shown in Fig. 2, iNKT cells in the presence of citrulline exposed to THP1 ± αGalCer present significant metabolic changes as measured by Seahorse assay (Supp Fig. 5a–h). The absence of citrulline also impairs antigen-induced IFN-γ release (Fig. 4f). Citrulline is transported by LAT-1 and catabolised by ASS1, an enzyme which infers resistance to a low arginine microenvironment (Supp Fig. 5g). After cross-talk with CD1d + AML (THP-1), ASS1 expression is upregulated in freshly isolated iNKT cells (Fig. 5a, and Supp Fig. 5h–j) with concurrent increased enzyme activity (Fig. 5b). Minimal change in the expression of other enzymes within the arginine metabolism pathway was seen (Arginase 1—ARG1, ARG2, Ornithine Transcarbamylase—OTC, Argininosuccinate Lyase—ASL) (Supp Fig. 5k). Confirming these findings ASS1 enzyme expression is similarly increased in murine iNKT cells within the AML environment in vivo (Fig. 5c–e).

### CD1d-iTCR-dependent cross-talk induces AML apoptosis

**Fig. 6:**
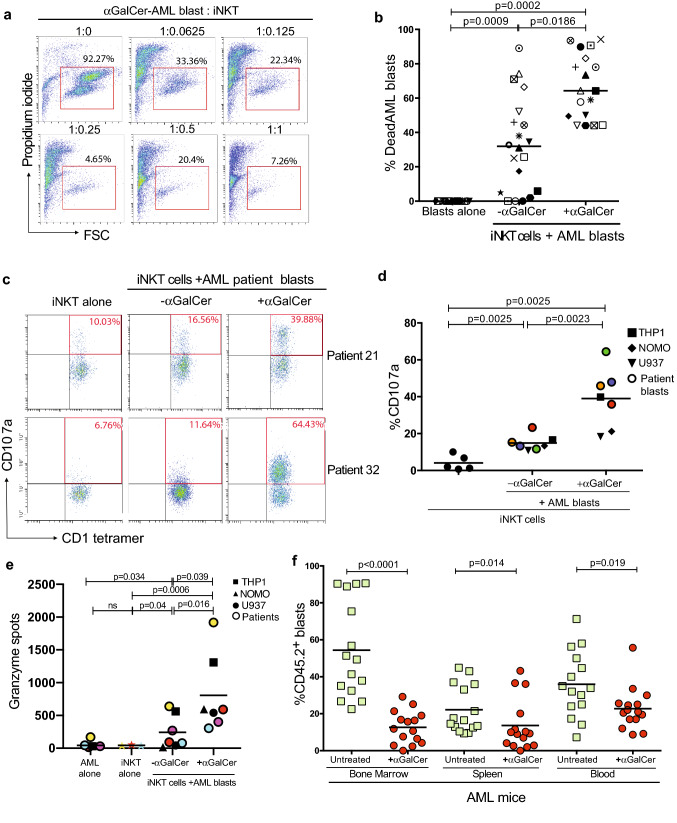
iNKT cell activation induces AML cell death. **a **αGalCer (100 ng/ml) enhances iNKT cell line cytotoxicity against AML blasts after 72 h of culture. Representative flow cytometry of n = 18 patients, gated on propidium iodide staining versus forward scatter (FSC) of AML blasts. **b** Pooled analysis demonstrating αGalCer (100 ng/ml) enhances iNKT cell line cytotoxicity against AML blasts (n = 18 donors) as measured by flow cytometry. (1iNKT:4AML blasts). *P* value determined by paired t-test. **c** Representative flow cytometry of iNKT cells, gating on CD1d tetramer versus CD107a, after iNKT co-culture with AML blasts. Two representative patients of n = 7 total. **d** iNKT cells undergo degranulation, exemplified by CD107a upregulation, following αGalCer (100 ng/ml) induced cross-talk with AML (n = 3 cell lines and n = 4 AML patient samples) for 72 h. Each dot is the mean of duplicates. **e** Increased granzyme B expression in iNKT cell lines after 72 h of cross-talk with n = 3 AML cell lines and n = 3 AML patients as measured by ELISPOT. Each dot is the mean of duplicates. **f** iNKT cell activation with αGalCer (2 μg /mouse on days 5 and 10 iv) in vivo leads to a reduction in MLL-AF9 leukaemia burden in the bone marrow, spleen, and blood (n = 15 mice untreated mice and n = 15 mice treated with αGalCer). AML blasts (CD45.2) were measured by flow cytometry. Data from n = 3 individual experiments. *P* value determined by unpaired t-test

Having demonstrated that iNKT cells are functional in the AML niche we investigated the impact of iNKT cells on AML disease burden. iNKT cells are cytotoxic to blasts on antigen presentation (Fig. 6a, b, Supp Fig. 6a) in a CD1d-CD40-dependent manner (Supp Fig. 6b, c). Blockade of CD1d prevents IFN-γ release (Supp Fig. 6d). iNKT cells undergo degranulation exemplified by CD107a upregulation (Fig. 6c and d), and granzyme release (Fig. 6e). The result is induction of AML blast apoptosis characterised by Caspases 9,3 and PARP cleavage (Supp Fig. 6e, Supp Fig. 7a). In vivo activation of iNKT cells with αGalCer similarly leads to a significant reduction in CD1d + AML (Supp Fig. 7b) burden in the blood, spleen, and bone marrow (Schematic Figs. 3e and 6f).

### iNKT rescue antigen-specific T cell proliferation in other myeloid-derived immunosuppressive microenvironments

Physiological antigen-specific conventional T cell expansion in AML patients or patients with solid cancers is often incapable of controlling cancer progression. Clinical trials of T cell therapies remain suboptimal outside of B-cell malignancies, due to poor T cell persistence and activation and the impact of MDSCs [26, 27]. As we show that iNKT cells can function within a low arginine microenvironment we investigated their role as an adjunct to T cell immunotherapy in two further models. Co-culture of allo-antigen-driven T cells with AML blasts led to a reduction in T cell proliferation, which was restored in the presence of activated iNKT cells (Supp Fig. 7c). These findings are recapitulated in vivo with AML-bearing mice (MLL-AF9) similarly undergoing a rescue of T cell frequency in the spleen and bone marrow after αGalCer treatment (Supp Fig. 7d).

**Fig. 7:**
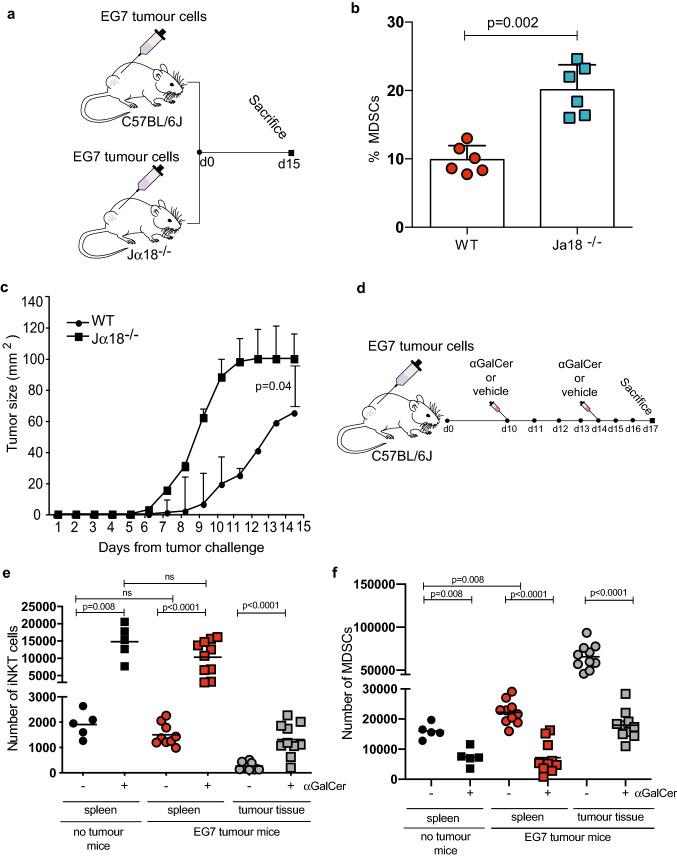
iNKT cells control MDSC numbers in an EG7 lymphoma model. **a** Schematic depicting experimental design. C57BL/6J or Jα18^−/−^ mice were engrafted with EG7 cells subcutaneously. 15 days later mice were sacrificed. **b** MDSCs are increased in the spleens of Jα18^−/−^ mice (n = 6) compared to wild-type (WT; n = 6), as measured by flow cytometry. *P* value determined by unpaired t-test. **c** Lymphoma (EG7) growth is faster in (n = 6) Jα18^−/−^ mice compared to wildtype (WT; (n = 6). *P* value determined by unpaired t-test. **d** Schematic depicting experimental design. C57BL/6J were engrafted with EG7 cells subcutaneously. Mice were treated with iv αGalCer 2 μg /mouse or vehicle on days 10 and 14, before being sacrificed on day 17. **e** iNKT cells expand in the tumours and spleens of EG7 lymphoma-bearing following αGalCer (n = 10 mice per group, vs n = 5 no tumour-bearing mice controls). Data of two individual experiments. *P* value determined by unpaired t-test. **f** MDSCs are reduced in the tumours and spleens of EG7 lymphoma-bearing mice following αGalCer 2 μg /mouse (n = 10 mice per group, vs n = 5 no tumour-bearing mice controls). Data two individual experiments. *P* value determined by unpaired t-test

MDSCs and AML blasts share phenotypic and immunosuppressive similarities [9–11]. Therefore, we investigated the function of iNKT in a second immunocompetent system, using a syngeneic lymphoma model that induces CD1d positive MDSCs in vivo (Supp Fig. 7e, f). EG7 lymphoma cells were engrafted into wild-type and iNKT cell-deficient mice (Jα18^−/−^ mice) (Fig. 7a). Jα18^−/−^ mice have a relative increase in MDSCs (Fig. 7b) and developed larger tumours (Fig. 7c) compared to WT mice. To evaluate the potential role of iNKT activation in MDSC control, EG7 tumour-bearing mice were treated with αGalCer (Fig. 7d) leading to an increase in iNKT cell frequency in the tumours and spleens (Fig. 7e), and associated IFN-γ release into the serum (Supp Fig. 7g), with a reduction in the MDSCs in these tissues (Fig. 7f). We confirmed iNKT cells within the EG7 tumour microenvironment express ASS1, regardless of αGalCer treatment (Supp Fig. 8a, b).

**Fig. 8:**
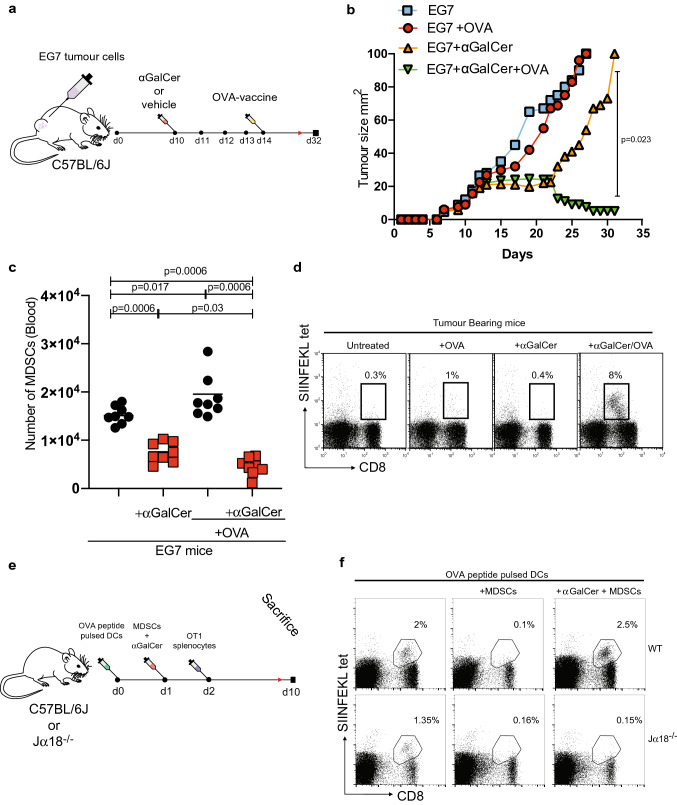
iNKT cells control MDSC numbers in an EG7 lymphoma model. **a** Schematic depicting experimental design. C57Bl/6J mice were engrafted with EG7 cells subcutaneously. Mice were treated with iv αGalCer 2 μg /mouse or vehicle on day 10. Two groups were treated with OVA-vaccine on day 14. **b** Lymphoma (EG7) growth is inhibited in OT-1 mice treated with OVA and 2 μg /mouse αGalCer (n = 8 mice per group). *P* value determined by unpaired t-test. **c** EG7 lymphoma-bearing mice (C57BL/6J), treated with OVA-vaccine and 2 μg /mouse αGalCer (n = 8 mice per group), have a corresponding decrease in the total number of MDSCs in the blood. *P* value determined by unpaired t-test. **d** Representative flow cytometry plot, gated on CD8 versus SIINFEKL-tetramer positive T cells, demonstrating increased SIINFEKL antigen-specific T cells in the blood of EG7 lymphoma-bearing mice (n = 8) treated with OVA-vaccine and 2 μg /mouse αGalCer. **e** Schematic depicting experimental design. C57BL/6J or Jα18^−/−^ mice were administered OVA-peptide-loaded dendritic cells (DC) and adoptively transferred tumour-derived MDSCs. Following the administration of αGalCer (2 μg /mouse) and OT1 splenocytes iv mice were sacrificed on day 10. **f** Antigen-specific T cells expansion is inhibited by the lack of iNKT cells in Jα18^−/−^ mice adoptively transferred with tumour-derived MDSCs. Representative flow cytometry gating of SIINFEKL tetramer labelled T cells from the spleens. Representative data of two individual experiments

Next to evaluate the impact of iNKT activation on conventional T cell immunity, antigen-specific T cells were induced with OVA-peptide vaccine (Fig. 8a). Vaccine alone led to no significant control of tumour growth (Fig. 8b). However, combination with αGalCer led to a significant slowing of tumour growth (Fig. 8b), reduction of MDSC frequency in blood (Fig. 8c) and an enhanced OVA-specific T cell proliferation (Fig. 8d and Supp Fig. 8c).

To confirm the capacity of activated iNKT to overcome MDSC inhibition of conventional T cells, MDSCs from EG7 lymphoma-bearing mice were adoptively transferred into wild-type (WT) and Jα18^−/−^ naïve mice (Fig. 8e). MDSCs abolished in vivo expansion of adoptively transferred OT-I splenocytes following peptide priming (Fig. 8f and Supp Fig. 8d). In contrast, when the transfer of MDSCs was followed by an injection of αGalCer in Jα18^−/−^ mice, no expansion of adoptively transferred OT-I cells was observed (Fig. 8f, Supp Fig. 8d).

## Discussion

We demonstrated that iNKT cells function within the immunosuppressive environments created by AML or MDSCs to reduce myeloid numbers and enhance antigen-specific T cell responses. AML and MDSCs share a number of characteristics, not least their myeloid origin, but also the downregulation of HLA-DR and suppression of T cell proliferation. One of the most well-characterised suppressive mediators in MDSCs is the expression of ARG1, which catabolises arginine to deprive T cells [28, 29]. The regulation of ARG1 expression in MDSCs by IL-4, HIF-1α, and STAT3 is well established; however, the factors which regulate ARG2 in tumour cells have received less attention to date [30, 31]. Here, we show that serum amyloid A is produced by AML blasts and promotes blast viability through FPR2. Notably, SAA also regulates IDO expression in AML blasts [32]. SAA has been reported to regulate ARG1 and ARG2 in endothelial cells or macrophages, which may promote myeloid cell survival during infection and inflammation [33–35]

Previously, we and others have shown that although T cells can recognise a number of peptide antigens presented by AML blasts in the context of HLA molecules, the capacity to meaningfully boost these responses in clinical trials is challenging[14, 36]. The identification that CD1d is expressed on AML blasts provides an opportunity to use non-peptide-based strategies to target AML. The number of iNKT cells in different cohorts of AML patients may vary[37, 38]. The role of iNKT cells as critical to anti-leukaemia immune surveillance was highlighted in a study demonstrating that the failure of iNKT cells to reconstitute in AML patients post-T cell-depleted HLA-haploidentical haematopoietic stem cell transplant (HSCT) correlated with relapse [39]. To date no clinical trials of iNKT cells against AML have taken place. The use of exogenous glycolipids, namely αGalCer, has the potential to translate our findings for therapeutic benefit. αGalCer remains an attractive therapeutic as it is relatively cheap to synthesise and has already completed Phase I and II clinical trials in adult solid tumours, demonstrating an excellent safety profile. We have shown αGalCer is capable to induce a number of critical effects including an increase of iNKT numbers, activation-induced IFN-γ release, a direct iNKT-derived cytotoxicity against AML blasts in vivo, and restoration of T cell function—expanding the rationale from previous in vitro reports [40, 41]. Interestingly, the potential of αGalCer-induced iNKT cell expansion has been harnessed in a hybrid fashion through the preclinical development of anti-CD19 iNKT CAR-T cells for lymphoma [42]. As AML blasts can directly present αGalCer to iNKT cells, the need for a loaded-dendritic cell vaccine or allogeneic approaches is also circumvented making the drug readily deliverable even to fragile AML populations such as children and the elderly.

We find that iNKT cells can function within the low arginine conditions created by AML, that is hostile to T cells. Although T cells require arginine for proliferation and activation, we show that iNKT cells do not express the cellular machinery for arginine catabolism (Arginase 1 or Arginase 2) but instead upregulate amino acid uptake via SLC7A5 (LAT-1) which feed forward into the ASS1 pathway. The binding partner of SLC transporters, CD98, has previously been reported on murine iNKT cells [43]. These findings help explain how iNKT cells can also function to abolish ARG1 + MDSCs that suppress T cells in the setting of influenza [44]. As such iNKT cells have a seemingly unique adaptation to the amino acid metabolic microenvironment of tumours, identifying a new niche functionality for these relatively rare immune cells. We highlight that αGalCer activation of iNKT cells leads to a secondary enhancement of antigen-specific T cell immunity not only in AML but in solid cancer—a hitherto unutilised finding. Although as a single agent αGalCer-iNKT responses against solid tumours in clinical trials have been disappointing, our data suggest a better strategy is to combine αGalCer-induced iNKT activation as an adjuvant to T cell immunotherapies. We show that iNKT activation abrogates the expansion and immunosuppressive activity of tumour-associated MDSCs in immunocompetent murine models and allows antigen-specific T cells to eradicate tumour burden. In many respects, this mimics the multi-cellular immunological response that is physiologically generated on immune activation against foreign pathogens [45]. Targeting of MDSCs remains a major challenge in the era of cellular immunotherapy, due to the heterogeneity of MDSC phenotype both within patients and across diseases. Although MDSC depletion with cytotoxic chemotherapy remains an approach used prior to CAR-T/ adoptive T cell administration, the short-lived effectiveness of such an approach likely contributes to the failure of T cell therapies in solid cancers [46]. As iNKT cells are produced endogenously by patients and circulate both through the blood and tumour compartments, iNKT cells could be activated in patients by treatment with αGalCer or ex vivo using apheresed and expanded products. Thus, iNKT cell therapy could be given alongside other immune therapies, such as CAR-T, and provide a sustainable, translatable and low toxicity approach to enhance immunotherapy.

## Key points

iNKT cells cross-talk with AML blasts via CD1d-dependent signalling, resulting in AML apoptosis and reduction of leukaemic burden in vivo. iNKT upregulate LAT-1/ ASS1 in low arginine environments leading to activation of antigen-specific T cells and tumour clearance.

## Supplementary Information

Below is the link to the electronic supplementary material.

Supplementary file1 (PDF 250 KB)

Supplementary file2 (PDF 655 KB)

Supplementary file3 (PDF 2319 KB)

Supplementary file4 (PDF 2721 KB)

Supplementary file5 (PDF 758 KB)

Supplementary file6 (PDF 5980 KB)

Supplementary file7 (PDF 780 KB)

Supplementary file8 (PDF 3595 KB)

Supplementary file9 (PDF 1364 KB)

Supplementary file10 (PDF 86 KB)
