# Supplementary material for: Invariant NKT cells metabolically adapt to the acute myeloid leukaemia environment
Source: Cancer Immunol Immunother. 2022 Aug 13;72(3):543–60. doi: 10.1007/s00262-022-03268-4 (PMC9947083; doi:10.1007/s00262-022-03268-4)
Supplement: Supplementary file 1 — Supplementary file1 (PDF 250 KB) [file 262_2022_3268_MOESM1_ESM.pdf]

Supplementary Methods

Cell lines

AML cell lines THP-1, U937, KG1a, and NOMO were obtained from DSMZ. All cell lines were validated for authenticity by DNA Short Tandem Repeats in line with American National Standards Institute ASN-0002-2011 (Northgene). Cells were routinely cultured in RPMI-1640 (Sigma) supplemented with 10% v/v heat-inactivated foetal bovine serum (FBS), glutamine (2mM), sodium pyruvate (1mM) and Penicillin-Streptomycin (100U) (R10%) using T-75 flasks kept in a humidified air atmosphere with 5% CO_2 at 37°C. For effects of citrulline deprivation cells cultured in RPMI-1640 supplemented with 10% v/v dialyzed FBS (Thermo Fisher Scientific). For arginine free culture media, RPMI-1640 for SILAC (Thermo Fisher Scientific) which is deficient in L-arginine was used. 75% of low arginine media was made by combining arginine free culture media with routine culture media at a ratio of 3:1.

Generation of human dendritic cells

PBMCs were isolated by Lymphoprep gradient centrifugation. Monocytes were isolated using anti-CD14 magnetic beads (MACS, Miltenyi Biotec). Dendritic cells (DCs) were then generated by culturing monocytes with 50ng/ml GM-CSF and 1000 U/ml IL-4 (Peprotech) for 5 days in a 6 well plate.

Cell culture assays

AML patients' blasts ($1x10^5$) were incubated in R10% with SAA (10μg/ml; Peprotech) for 72hours. Where indicated anti-TLR2 (clone TL2.2), -TLR4 (clone HTAR5), -FPR2 (clone K102B9) antibodies (10μg/ml; Biolegend) were added to cultures 4 hours in advance to block the respective receptors. Similarly anti-CD1d (25μg/ml, clone 541, BD Biosciences) or anti-CD40 (10μg/ml, clone 5C3, Biolegend) antibodies were added as indicated for 1 hour and then co-cultured with αGalCer (100ng/ml) and iNKT cells ($0.25x10^5$) in R10% for 72hours. Supernatants were harvested to determine arginine concentrations or IFN-γ release by ELISA and cell pellets of sorted iNKT cells collected for subsequent analyses. 10μM of p38 MAPK inhibitor SB203580 (Cell Signalling Technologies) was added 1hour

before further treatments in relevant experiments. LAT-1 inhibitor JPH203 (0.125mM) was added to cell cultures for 72hours in relevant experiments

Arginine ELISA

The concentration of arginine in human plasma, murine serum or culture supernatants was quantified using a competitive enzyme linked immunoassay (K7733, Immunodiagnostik, Germany) according to the manufacturers' instructions. In brief, the assay uses a competitive enzyme immunoassay in which L-arginine is derivatized from samples and competes with an L-arginine-tracer for binding of polyclonal antibodies, in the microtiter wells. The concentration of the tracer-bound antibody is inversely proportional to the L-arginine concentration in the samples.

Cytokine detection assays

For detection of multiple cytokines in plasma or culture supernatants LegendPlex multiplex flow cytometry for a Th cytokine panel and murine anti-virus response panel (BioLegend) were used. The concentration of TNF-α, IL-1β, human and mouse IFN-γ (BioLegend), GM-CSF (R&D systems), SAA (Invitrogen), IL-13 and CRP (eBioscience) was measured by ELISA according to the manufacturer's instructions.

Confocal Microscopy

To determine SAA in AML blasts $1.0x10^5$ cells were stained with unconjugated anti-SAA antibodies (Abcam), followed by FITC-conjugated anti-rabbit antibody (Santa Cruz). ASS1 expression in iNKT cells, $1.0x10^5$ cells were stained with a FITC conjugated anti-human TCR Vα24 antibody (Miltenyi Biotech). Stained cells were spun unto 13-mm glass coverslips at 1000 rpm for 5 minutes then fixed in 4% paraformaldehyde (Sigma) in PBS. Fixed cells were washed three times in 1x PBS and permeabilised with 0.5% Triton-X100 for 1 minute. Permeabilised cells were blocked in PBS containing 5% goat serum

and 0.05% Tween-20 then incubated with the primary antibody overnight. Cells were washed and incubated with a secondary antibody for 2hours. Stained cells were mounted onto glass slides in a single drop of SlowFade™ Gold Antifade Mountant with DAPI and examined using a Zeiss LSM 780 fluorescence confocal microscope. Analysis was performed on acquired images using ZEN software suite (Carl Zeiss Microscopy).

Granzyme B ELISPOT

Granzyme B release was measured by ELISPOT (R&Dsystems) according to manufacturer's instructions. In brief, 96 well PDVF plates coated with a Granzyme B polyclonal antibody. $1X10^5$ patient AML blasts and AML cell lines were plated in the presence of $0.25x10^5$ iNKT cells. After 24hours the plates were washed and biotinylated detection antibody added. Following washing, avidin-HRP solution was added and the plate incubated for 45minutes. Following a further wash, substrate solution was added, and left to develop until spots developed, at which point the reaction was terminated by washing, and the plate was left to dry. Images of spots were taken using a Bioreader 5000fγ (Biosys).

Immunoblotting

Following cell lysis of isolated iNKT cells, in RIPA buffer (ThermoFisher Scientific) containing c0mplete™ protease and phosSTOP™ phosphatase inhibitors (Roche Applied Science, Indianapolis, IL), equal amounts of protein were loaded in 30µL of Laemmli sample buffer (BioRad) onto 4-20% Criterion™ Tris-Glycine eXtended (TGX) stain-free precast gels (BioRad) and electrophoresed in a Criterion™ Cell (Biorad) powered by a PowerPac™ basic power supply at 200V for 1 hour. Protein gels were blotted using the Trans-Blot Turbo transfer apparatus unto 0.22µm PVDF MIDI transfer membranes (BioRad). Membranes were incubated with gentle agitation in blocking buffer consisting of 5% BSA in PBS for 1

hour at room temperature. Membranes were incubated overnight at 4°C with gentle agitation with rabbit anti-human LAT-1(#5347S), Caspase9 (clone E5Z7N), Caspase 3, PARP (clone 46D1), phospho-p38(#9215S), p38(#9212S), ARG1(clone D4E3M),ARG2 (clone D9J1N), beta actin (#8457S) (Cell Signalling Technologies), rabbit anti-human ASS1(#HPA020896), OTC(HPA000243), and ASL (Abcam #ab97370). HRP-conjugated secondary antibodies, goat anti-rabbit (Cell Signalling), and sheep anti-mouse (GE Healthcare) were used for blots, which were developed with ECL substrate (BioRad) and exposed on Kodak film or imaged on a Chemidoc MP system (BioRad).

Transmission Electron microscopy

AML cell lines were cultured with αGalCer (100ng/ml) and iNKT cells for 72hours as above. AML cell lines were isolated by negative selection. Following harvesting they were fixed in 2.5% glutaraldehyde followed by 1% osmium tetroxide. The samples were dehydrated through ethanol and embedded in propylene oxide/resin mixture at 60°C for 16h prior to sectioning at 80 nm in thickness and placement on 300 mesh copper slot grids for examination by transmission electron microscopy.

Cellular Bioenergetics

AML cell lines THP1 or KG-1a were pulsed ± α-GalCer (100ng/ml) for 4 hours before being washed and co-cultured with iNKT cells at an effector to target ratio of 0.25:1 for 24 hours. Where indicated 100ng/ml citrulline was added to culture conditions. iNKT cells were positively selected with anti-human iNKT cell microbeads according to manufacturer's instructions and immobilised onto CellTak-coated (Corning) Agilent Seahorse XFe96 cell culture microplates (V3-PS, Agilent Technologies) at a density of 2.5×10^5 cells/well in 180µl/well RPMI (R8755, Sigma-Aldrich) supplemented with 5mM HEPES (pH 7.4), 5mM glucose, 2mM L-Glutamine and 1 mM sodium pyruvate. Following a 1-hour incubation at 37°C under air (non-CO_2 incubator), and calibration of the XFe96 sensor cartridge, the cell culture microplate was transferred to the Seahorse XFe96 extracellular flux analyzer (controlled

at 37°C). Baseline oxygen consumption rates (OCR) and extracellular acidification rates (ECAR) were measured for 4 cycles consisting of a 3-minute mix and 3-minute measure period before sequential addition of 2 µg/ml oligomycin, 3 µM BAM15, and a mixture of 2 µM rotenone plus 2 µM antimycin A to determine rates of ATP coupled respiration, maximal and spare respiratory capacities, and non-mitochondrial cellular oxygen uptake, respectively. Proton efflux rate was calculated from ECAR using the following formula: PER (pmol H^+/min) = ECAR (mpH/min) x BF (mmol H^+/L/pH) x Vol $_{XF\ microchamber}$ x Kvol; Kvol = 1.6, BF = 2.5 and Vol XF microchamber = 2.3. To establish glycolytic PER (glycoPER), PER were corrected for mitochondrial acidification (mitoPER) by multiplying mitochondrial OCR by the CO2 contribution factor (0.61). Coupling efficiency is determined as the amount of mitochondrial respiration that is coupled to ATP synthesis and calculated as the percentage of oxygen consumption inhibited by the ATP synthase inhibitor oligomycin from basal mitochondrial respiration (Formula - oligomycin sensitive OCR / basal mitochondrial OCR (basal OCR - non-mitochondrial OCR) x 100. ATP synthesis rates are estimated from both OCR and PER data using the relevant P/O (Mol of ATP generated per mol of oxygen consumed) ratios for glucose based on established methodology. [1,2]

Arginase activity

The activity of ARG2 present within AML blasts, was determined by measuring the conversion of arginine into urea. Patient AML blasts or cell lines were cultured with SAA (10µg/ml) in RPMI+10% FCS at 37°C, 5% CO_2. After 24 hours the supernatants were collected and the cells pelleted and lysed with 50 µl of buffer containing 0.1% Triton X-100, 5 µg pepstatin, 5 µg aprotinin and 5 µg antipaina. The samples were placed on a 37°C heat block for 30 minutes before centrifugation at 14,000 rpm and collection of the supernatants. To activate the arginase enzyme, buffer containing Tris-HCl (25 mM) and $MnCl_2$ (10 mM) was added and heated to 56°C for 10 minutes. L-arginine (0.5M, Sigma) was added and the samples were heated for 1 hour at 37°C. The hydrolysis of arginine was stopped with 800 µl of an acid solution mixture (H_2SO_4:H_3PO_4:H_2O, 1:3:7). The amount of urea produced was determined

using 9% α-isonitrosopropriophenone and compared to a standard curve with absorbance measured at 540nm.

ASS1 activity assay

The activity of ASS within iNKT cells was determined by measuring the catabolism of citrulline. iNKT cells were cultured with AML cells and αGalCer for 72hours. iNKT cells were positively selected with anti-human iNKT beads (Miltenyi) and lysed with 20 μl of lysis buffer (0.1% Triton X-100, 5 μg pepstatin, 5 μg aprotinin and 5 μg antipaina) on ice for 20 minutes. To 20μl of each sample lysate supernatant, 10µl of L-citrulline (4mM, pH 7.5), 10µl of L-aspartic acid (4mM, pH 7.5), 10µl of $MgCl_2$ (6mM), 10µl of ATP (4mM, pH 7.5), 40µl of Tris-HCl (20mM) was added, before placing on a 37°C heat block for 90 minutes to allow the enzyme reaction to occur. The hydrolysis of citrulline was stopped with 80 μl of an acid solution mixture (3:1 mix of phosphoric acid and sulphuric acid) and 20μl of 3% 2,3butanedione monoxime, followed by vortexing and incubation at 95C for 30 minutes. The amount of citrulline at the end of the assay was determined by comparison to a standard curve with absorbance measured at 490 nm.

Untargeted metabolomics applying ultra high performance liquid chromatography-mass spectrometry

To extract polar metabolites from isolated iNKT cells, the cell pellets were mixed with 500 µL 2:1:1 acetonitrile:methanol:water (all solvents were LC-MS grade, VWR) followed by two rounds of the following procedure: vortex (30 s), freeze on dry ice, thaw on wet ice. Samples were centrifuged (13,000-g, 4°C, 20 min) and 375 µL of supernatant was removed for each sample. A further 75 µL of supernatant was removed from each sample and pooled to create a pooled QC sample. The QC sample was mixed by vortexing (30 s) and split into multiple 375 µL aliquots. An extraction blank was created using the same extraction procedure in the absence of cells. All biological and QC samples and the extract blank were dried in a SpeedVac sample concentrator (Thermo Scientific) and stored at -80°C until analysis. Prior to analysis, 80 µL of 3:1 acetonitrile: water was added to dried metabolite extracts

and then vortexed (30 s), centrifuged (13,000- *xg*, 4°C, 20 min) and 60 µL of the supernatant loaded into low volume HPLC vials (VI-04-12-02RVG 300µL Plastic, Chromatography Direct, UK).

Samples were analysed as previously described, applying ultra high performance liquid chromatography-mass spectrometry (UHPLC-MS).[3,4,2] Samples were maintained at 4°C and analysed using a Dionex UltiMate 3000 Rapid Separation LC system (Thermo Fisher Scientific, MA, USA) coupled with a heated electrospray Q Exactive Focus mass spectrometer (Thermo Fisher Scientific, MA, USA). Mobile phase A consisted of 10 mM ammonium formate and 0.1% formic acid in 95% acetonitrile/water and mobile phase B consisted of 10 mM ammonium formate and 0.1% formic acid in 50% acetonitrile/water. Flow rate was set for 0.50 mL.min-1 with the following gradient: t=0.0, 1% B; t=1.0, 1% B; t=3.0, 15% B; t=6.0, 50% B; t=9.0, 95% B; t=10.0, 95% B; t=10.5, 1% B; t=14.0, 1% B, all changes were linear with curve = 5. The column temperature was set to 35 °C and the injection volume was 2 µL. Data were acquired in positive and negative ionisation modes separately within the mass range of 70 – 1050 m/z at resolution 70,000 (FWHM at m/z 200). Ion source parameters were set as follows: Sheath gas = 53 arbitrary units, Aux gas = 14 arbitrary units, Sweep gas = 3 arbitrary units, Spray Voltage = 3.5kV, Capillary temp. = 269 °C, Aux gas heater temp. = 438°C. Data dependent MS2 in 'Discovery mode' was used for the MS/MS spectra acquisition using following settings: resolution = 17,500 (FWHM at m/z 200); Isolation width = 3.0 m/z; stepped normalised collision energies (stepped NCE) = 25, 60, 100%. Spectra were acquired in three different mass ranges: 50 70 – 200 m/z; 200 – 400 m/z; 400 – 1000 m/z. A Thermo ExactiveTune 2.8 SP1 build 2806 was used as instrument control software in both cases and data were acquired in profile mode. Quality control (QC) samples were analysed as the first ten injections and then every seventh injection with two QC samples at the end of the analytical batch. Two blank samples were analysed, the first as the 6th injection and then the second at the end of each batch.

Raw data acquired in were converted from the instrument-specific format to the mzML file format applying the open access ProteoWizard (version 3.0.11417) msconvert tool (Kesnner D et al. Bioinformatics. 2008). During this procedure, peak picking and centroiding, were achieved using vendor algorithms. Deconvolution was performed with XCMS software according to the following settings of Min peak width (4); max peak width (30); ppm (12); mzdiff (0.001); bw (0.25); mzwid (0.01).[53] A data matrix of metabolite features (*m/z*-retention time pairs) vs. samples was constructed with peak areas provided where the metabolite feature was detected for each sample.

A quality assurance and quality control (QA/QC) assessment was performed to measure drift across retention time, *m/z* and signal intensity and identify potential outliers.[5] The first five QCs were used to equilibrate the analytical system and were subsequently removed from the data before the data was analysed. Principal Components Analysis (PCA) was performed to assess the technical variability (measured by the replicate analysis of a pooled QC sample) and biological variability as part of the quality control process. Prior to PCA missing values in the data were replaced by applying k nearest neighbour (kNN) missing value imputation (k = 5) followed by probabilistic quotient normalisation (PQN) and glog transformation prior to data analysis.

The data from the pooled QC samples were applied to perform QC filtering. For each metabolite feature detected QC samples 1-5 were removed and the relative standard deviation and percentage detection rate were calculated using the remaining QC samples. Blank samples at the start and end of a run were used to remove features from non-biological origins. Any feature with an average QC intensity less than 20 times the average intensity of the blanks were removed. Any sample with >50% missing values was excluded from further analysis. Metabolite features with a RSD > 30% and present in less than 90% of the QC samples were deleted from the dataset. Features with a <50% detection rate over all samples were also removed.

Putative annotation of metabolites or metabolite groups was performed by applying the PUTMEDID-LCMS workflows operating in the Taverna workflow environment.[6] We applied 12 ppm mass error and a retention time range of 2 s in feature grouping and molecular formula and metabolite matching. As different metabolites can be detected with the same accurate *m/z* (for example, isomers with the same molecular formula), multiple annotations could be observed for a single detected metabolite feature. Also, a single metabolite could be detected as multiple molecules, particularly as a different type of ion (e.g., protonated and sodiated ions). All molecules were annotated according to guidelines for reporting of chemical analysis results, specifically to Metabolomics Standards Initiative level 2.[7] Data were normalised applying probabilistic quotient normalisation (PQN) and glog transformation prior to statistical data analysis. 'Within-subjects repeated measures ANOVA' was applied to identify statistically significant changes. Pathway enrichment analysis using all annotated metabolites was performed applying the Enrichment Analysis module in MetaboAnalyst.[8]

Supplementary References

1. Mookerjee S, Gerenscser A, Nicholls D, Brand M. Quantifying intracellular rates of glycolytic and oxidative ATP production and consumption using extracellular flux measurements. *J Biol Chem* 2017; **292**(17):7189-7207
2. Mookerjee S, Goncalves R, Gerenscser A, Nicholls D, Brand M. The contibutions of respiration and glycolysis to extracellular acid production. *Biochim Biophys Acta* 2015; **1847**(2):171-181
3. D'Elia RV, Goodchild SA, Winder CL, et al. Multiple metabolic pathways are predictive of ricin intoxication in a rat model. *Metabolomics* 2019; **15**(7): 102.
4. Smith CA, Want EJ, O'Maille G, Abagyan R, Siuzdak G. XCMS: processing mass spectrometry data for metabolite profiling using nonlinear peak alignment, matching, and identification. *Anal Chem* 2006; **78**(3): 779-87.
5. Broadhurst D, Goodacre R, Reinke SN, et al. Guidelines and considerations for the use of system suitability and quality control samples in mass spectrometry assays applied in untargeted clinical metabolomic studies. *Metabolomics* 2018; **14**(6): 72.
6. Brown M, Wedge DC, Goodacre R, et al. Automated workflows for accurate mass-based putative metabolite identification in LC/MS-derived metabolomic datasets. *Bioinformatics* 2011; **27**(8): 1108-12.
7. Sumner LW, Amberg A, Barrett D, et al. Proposed minimum reporting standards for chemical analysis Chemical Analysis Working Group (CAWG) Metabolomics Standards Initiative (MSI). *Metabolomics* 2007; **3**(3): 211-21.
8. Chong J, Xia J. MetaboAnalystR: an R package for flexible and reproducible analysis of metabolomics data. *Bioinformatics* 2018; **34**(24): 4313-4.

Supplementary Figure Legends

Supplementary Figure 1. SAA signalling in AML blasts

a) Plasma arginine concentrations in AML patients (n=32) at diagnosis compared to healthy donors (n=33), as measured by ELISA. Red symbol = Blood samples. Yellow symbol = Bone Marrow samples. p value determined by unpaired t-test. b) Serum arginine concentrations in MLL-AF9 AML (n=22) bearing mice at the termination of experiment (day 17), compared to no AML mice (n=7) as measured by ELISA. p value determined by unpaired t-test. Representative data of duplicate experiments. c and d) Cytokine ELISA in the plasma from n=18 newly diagnosed AML patients prior to treatment, compared to levels in n= 10 healthy donors. p value determined by unpaired t-test. e) SAA (10μg/ml) leads to an increase in viable AML blasts sorted from the blood and bone marrow, after 72hours as determined by flow cytometry. Representative of n=5 individual donors. f) Increased viability of AML blasts cultured with SAA (10μg/ml) for 72hours as determined by propidium iodide uptake measured by flow cytometry. n=8 individual donors shown. Each dot is the mean of duplicates. p value determined by paired t-test.

Supplementary Figure 2. iNKT interactions with AML blasts

a) Percentage of AML cell lines (n=6) and AML blasts from patients (n=13) expressing TLR2, TLR4, and FPR2 as determined by flow cytometry. b) AML blasts were cultured with SAA (10μg/ml) for 72hours in the presence of anti-TLR2, TLR4, and FPR2 blocking antibody (10μg/ml) for 72hours. The percentage of viable cells relative to untreated controls was determined by flow cytometry. Representative data of n=4 individual donors. c) AML blasts were cultured with SAA (10μg/ml) for 72hours in the presence of anti-TLR2, TLR4, and FPR2 blocking antibody (10μg/ml) for 72hours. The percentage of viable cells relative to untreated controls was determined by flow cytometry. Each dot is the mean of duplicate samples from three individual experiments. p value determined by paired t-test. d) Treatment of AML blasts with SAA (10μg/ml) for 72hours leads to increased IL-1β release into culture supernatants, as

measured by ELISA (n=15). Each dot is the mean of duplicates. p value determined by paired t-test. e) IL-1β is increased in the supernatants of AML patients' blasts treated with SAA (10μg/ml) for 72 hours, as measured by ELISA. Each dot is the mean of duplicates. p value determined by paired t-test. f) AML blasts (1x10^{6}) were treated with SAA (10μg/ml) for 72hours. SAA treatment leads to a reduction in supernatant arginine (n=12), as measured by ELISA. Each dot is the mean of duplicates. p value determined by paired t-test.

Supplementary Figure 3. iNKT cells are activated by AML blasts

a) Representative flow cytometry gating strategy for iNKT detection using anti-human CD1d tetramer. b) Representative flow cytometry and pooled data showing iNKT purity after cell sorting, using anti-human CD1d tetramer (n=10) c) Representative flow cytometry gating strategy for CD1d and CD40 expression on AML blasts. d) Expression of CD1d and CD40 on AML blasts from patients (n=27) and cell lines (n=6) as detected by flow cytometry. e) αGalCer (100ng/ml) presentation by AML cell lines induces freshly isolated primary iNKT cell proliferation, after 96hours. Flow cytometry plot showing CFSE dilution in proliferating iNKT cells. (f) A representative OCR trace from iNKT cell lines ± THP1 or KG1a ± αGalCer (100ng/ml pulsed for 4 hours), baseline respiration was assessed for 4 measurement cycles (each cycle consisted of a 3 min mix and 3 min measure period) prior to injection of oligomycin, BAM15 and a mixture of rotenone plus Antimycin A, to establish rates of ADP phosphorylation, maximal respiratory activity, and non-mitochondrial oxygen consumption, respectively. (g) Proton efflux rate (PER) from iNKT cell lines ± THP1 or KG1a ± αGalCer (100ng/ml pulsed for 4 hours) before and after oligomycin injection. (h) Mitochondrial oxygen consumption rates (OCR) in the absence and presence of oligomycin were assessed in iNKT cell lines ± THP1 or KG1a ± αGalCer (100ng/ml pulsed for 4 hours) to establish baseline rates of mitochondrial respiration (i) and rates of ATP-coupled mitochondrial respiration (g), respectively. No significant differences were observed. j) Estimated rates of glycolytic (ATPglyc) and mitochondrial (ATPmito) ATP synthesis in iNKT cell lines, were

calculated from baseline rates of glycoPER (total PER minus mitochondrial-linked PER) and ATP-coupled mitochondrial respiration. Mitochondrial OCR were corrected for non-mitochondrial respiration by subtracting OCR following rotenone and antimycin addition. Data are means ± SEM from 4 cell donors assessed from two independent microplates each containing 3-4 well replicates per donor. Statistical differences were assessed using one-way ANOVA with Fisher's LSD post hoc test.

Supplementary Figure 4. iNKT cells can be activated under low arginine conditions

a) Following co-culture, αGalCer (100ng/ml) presentation by AML blasts upregulates iNKT cell line intracellular IFN-γ, as measured by intracellular staining with flow cytometry after 72hours. iNKT cells are detected by CD1d tetramer staining. Data of n=2 experiments. b) Expression of CD69, FAS, CD154, and CD38 on sorted iNKT cell lines after co-culture with THP-1 cells in the presence of αGalCer (100ng/ml) for 72 hours, as measured by flow cytometry. Representative histogram plots and c) pooled geometric mean data from n=3 repeats. d) The concentration of arginine in the serum of C57BL/6J mice (n=8 per group) treated with pegylated recombinant human arginase (BCT-100; 10mg/kg) determined by colorimetric assay. Data of two individual experiments. p value determined by unpaired t-test e) Representative flow cytometry showing in vivo expansion of iNKT cells in the spleen of C57BL/6J mice treated with αGalCer, as detected by CD1d tetramer staining f) Treatment of C57BL/6J mice (n=8 per group) with pegylated recombinant human arginase (BCT-100) does not impair αGalCer-induced iNKT activation (2μg/mouse), measured by IFN-γ in the serum. Data of two individual experiments. p value determined by unpaired t-test. g) IFN-γ positive iNKT cell frequency in the blood is increased following αGalCer (2μg/mouse) administration to MLL-AF9 AML engrafted B6.SJL/J mice, measured by IFN-γ intracellular staining using flow cytometry. p value determined by unpaired t-test. h) iNKT activation with αGalCer (2μg/mouse) is unaffected by MLL-AF9 AML engraftment in B6.SJL/J mice, measured by IFN-γ in the serum. Data of two individual experiments. p value determined by unpaired t-test. i) No effect on the viability of primary iNKT cells after anti-CD3/CD28 antibody

stimulation in the presence of JPH203 (0.125mM) after 72 hours, measured by flow cytometry. Each dot is the mean of duplicates. p value determined by paired t-test.

Supplementary Figure 5. iNKT cells upregulate ASS on activation

a) A representative OCR trace from iNKT cell lines, baseline respiration was assessed for 4 measurement cycles (each cycle consisted of a 3 min mix and 3 min measure period) prior to injection of oligomycin, BAM15 and a mixture of rotenone plus Antimycin A, to establish rates of ADP phosphorylation, maximal respiratory activity, and non-mitochondrial oxygen consumption, respectively. b) Proton efflux rate (PER) from iNKT cell lines before and after oligomycin injection. c) Maximal respiratory capacity of iNKT cell lines, expressed as fold change between BAM 15-induced OCR and baseline OCR. d) Spare respiratory capacity of iNKT cell lines, calculated as the difference between BAM 15-induced OCR and baseline OCR, expressed as percentage of maximal respiration. Glycolytic proton efflux rates (glycoPER – Total PER minus mitochondrial-linked PER) in the absence and presence of oligomycin were probed in iNKT cell lines to establish baseline rates of glycolysis e) and compensatory glycolysis f), respectively. g) Estimated rates of glycolytic (ATPglyc) and mitochondrial (ATPmito) ATP synthesis in iNKT cell lines. h) Coupling efficiency of oxidative phosphorylation (i.e the rate of mitochondrial respiration coupled to ATP synthesis) in iNKT cell lines ± THP1 ± αGalCer, calculated as the percentage of mitochondrial OCR sensitive to oligomycin**.** 100ng/ml citrulline was added to cell cultures for 24hours throughout. Mitochondrial OCR were corrected for non-mitochondrial respiration by subtracting OCR following rotenone and antimycin addition. Data are means ± SEM from 1 cell donor assessed in one microplate containing 3-5 well replicates. Statistical differences were assessed using one-way ANOVA with Fisher's LSD post hoc test g) Schematic of citrulline catabolism pathway. h) Western blot demonstrating increased ASS

expression in healthy donor primary iNKT cells freshly sorted from healthy donor blood co-cultured with THP1 for 72hours, and re-purified. Actin is shown as a loading control. Representative data of n=3 individual experiments. i) Western blot demonstrating increased ASS expression in healthy donor primary iNKT cells freshly isolated from healthy donor blood, after cross-talk with THP1 (CD1d+ve) or KG1a (CD1d-ve) for 72hours and re-purified. Actin is shown as a loading control. No upregulation is seen after culture with CD1d negative cell line KG1a. Representative data of n=2 experiments j) Western blot demonstrating increased ASS expression in primary iNKT cells, after cross-talk with THP1 for 72hours in the presence or absence of 100ng/ml αGalCer, and re-purified. Actin is shown as a loading control. Representative data of n=2 experiments. k) Minimal expression of ARG1, ARG2, or OTC enzymes in isolated iNKT cell lines in the presence or absence of 100ng/ml αGalCer with THP-1 for 72hours. ASL remains unchanged. Western blot with actin shown as a loading control.

Supplementary Figure 6. iNKT cells are cytotoxic to AML blasts

a) Representative flow cytometry gating strategy demonstrating AML and iNKT populations after 72 hours of co-culture. Propidium iodide staining reveals viable iNKT cell lines as labelled by CD1d-tetramer (G1) and a reduction in viable AML blasts (G0) b) Addition of CD1d blocking antibody (25μg/ml,1hour) to INKT-AML co-cultures prevents iNKT cytotoxicity in the presence of (100ng/ml) αGalCer. n=4 patients. p value determined by unpaired t-test. c) Addition of CD40 blocking antibody (25μg/ml,1hour) to INKT-AML co-cultures prevents iNKT cytotoxicity in the presence of (100ng/ml) αGalCer n=4 patients. p value determined by unpaired t-test. d) Addition of CD1d blocking antibody (25μg/ml,1hour) to INKT-AML co-cultures prevents iNKT IFN-γ release in the presence of (100ng/ml) αGalCer. n=4 patients. p value determined by unpaired t-test. e) AML undergoes apoptosis following 48hour co-culture with iNKT cells. Western blot demonstrating PARP, Caspase 9, and Caspase 3 cleavage. Actin is shown as a loading control.

Supplementary Figure 7. iNKT cells restore T cell proliferation in the AML environment

a) Transmission electron micrographs of AML cell lines following culture with iNKT cell lines in the presence of (100ng/ml) αGalCer after 72hours. AML cells demonstrate features consistent with late apoptosis. b) AML blasts from MLL-AF9 mice are CD1d positive as demonstrated by flow cytometry staining. Data of two individual experiments. c) AML blasts suppress conventional T cell proliferation (n=4 donors) in a mixed leukocyte reaction assay (allogeneic T cells and dendritic cells), which is rescued by iNKT cell lines in the presence of 100ng/ml αGalCer, measured by flow cytometry after 96hours. Each dot is the mean of duplicates. p value determined by paired t-test. d) In vivo, iNKT activation with 2μg/mouse αGalCer rescues T cells proliferation in the spleen and bone marrows of AML bearing mice (n=10 MLL-AF9 mice) compared to no AML mice (n=6) measured by flow cytometry. Data of two individual experiments. p value determined by paired t-test. e) Representative flow cytometry of MDSCs from the tumours of EG7-tumour bearing mice, confirming GR-1, CD1d, and CD40 expression. f) CD1d and CD40 expression of MDSCs from the tumours of EG7-tumour bearing mice. Data of two individual experiments. g) iNKT activation by 2μg/mouse αGalCer is not inhibited by the (EG7) lymphoma microenvironment, measured by IFN-γ release into the serum. (n=10 mice per group, vs n=5 no tumour bearing mice controls). Data of two individual experiments. p value determined by unpaired t-test.

Supplementary Figure 8. iNKT cells upregulate ASS within the lymphoma microenvironment and restores antigen-specific T cell responses

a) Flow cytometry analysis from EG7 tumour engrafted mice. Following mechanical disruption of EG7 lymphomas ex vivo, iNKT cells were labelled with CD1d-tetramer. iNKT cells demonstrate ASS expression. Intracellular isotype antibody staining shown as a control. b) Pooled analysis of ASS expression in iNKT cells sorted from the EG7 tumours (n=10) engrafted in mice. Geometric mean of

intracellular staining for ASS is shown as detected by flow cytometry. c) Increased SIINFEKL antigen specific T cells in the blood of EG7 lymphoma bearing mice (n=8) treated with OVA-vaccine and αGalCer. Data from two individual experiments. p value determined by unpaired t-test. d) Antigen-specific T cells expansion is inhibited by the lack of iNKT cells in Jα18$^{-/-}$ mice adoptively transferred with tumour-derived MDSCs. SIINFEKL-tetramer labelled T cells from the spleens were detected by flow cytometry. Data of two individual experiments. p value determined by unpaired t-test.
