# Supplementary figures and images for: Invariant NKT cells metabolically adapt to the acute myeloid leukaemia environment
Source: Cancer Immunol Immunother. 2022 Aug 13;72(3):543–60. doi: 10.1007/s00262-022-03268-4 (PMC9947083; doi:10.1007/s00262-022-03268-4)

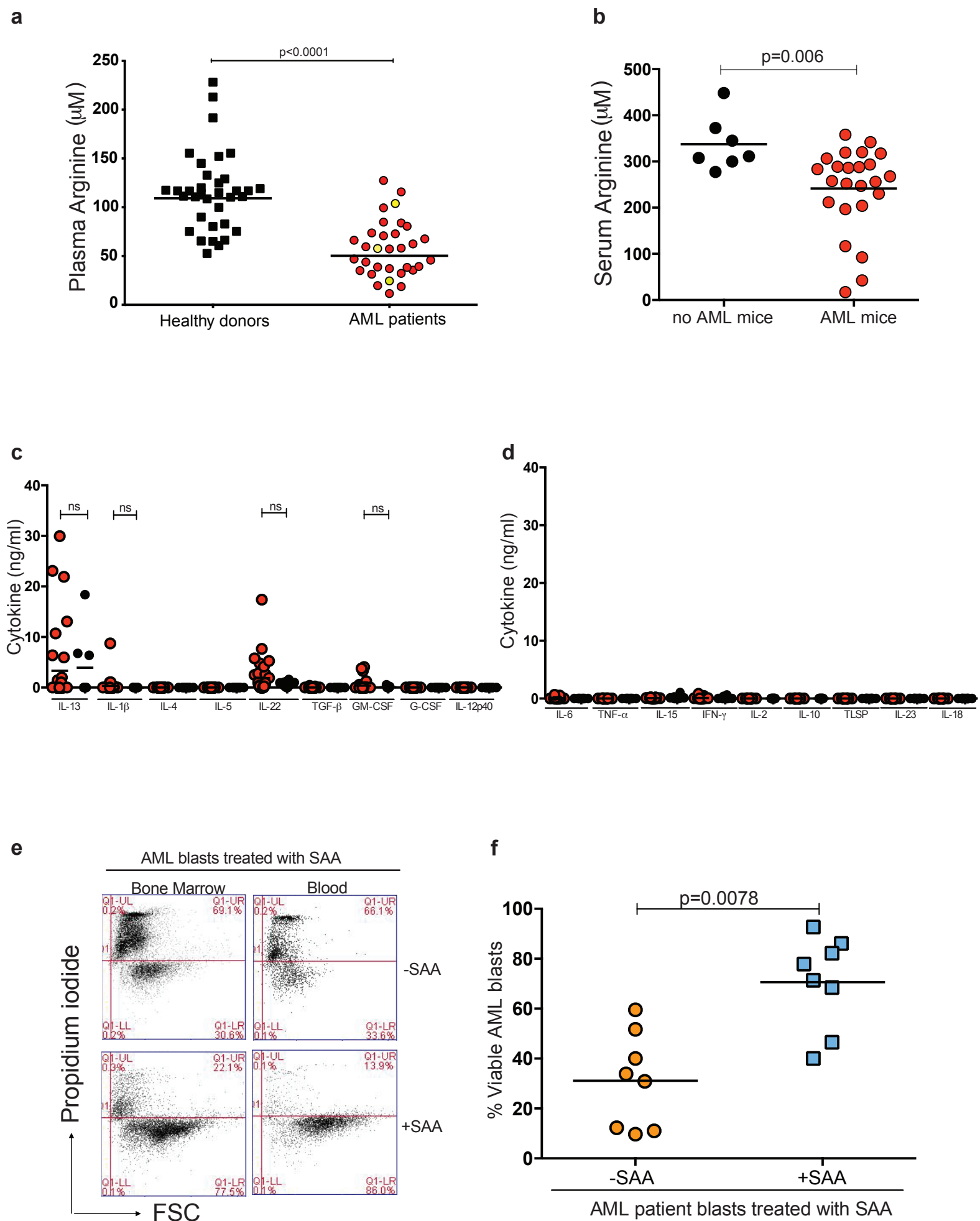

Supplement: Supplementary file 2 — Supplementary file2 (PDF 655 KB) [file 262_2022_3268_MOESM2_ESM.pdf]

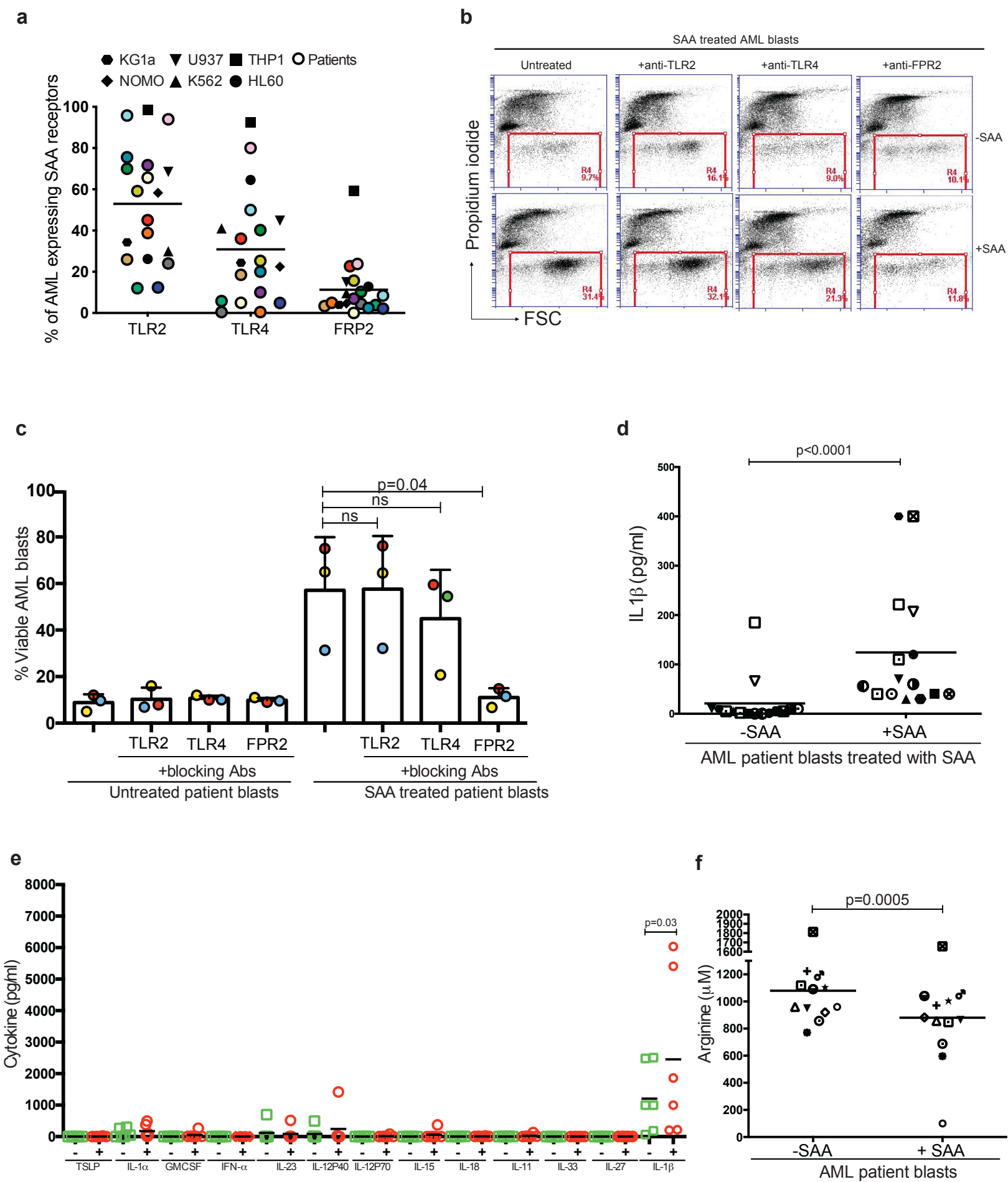

Supp Fig 2

Supplement: Supplementary file 3 — Supplementary file3 (PDF 2319 KB) [file 262_2022_3268_MOESM3_ESM.pdf]

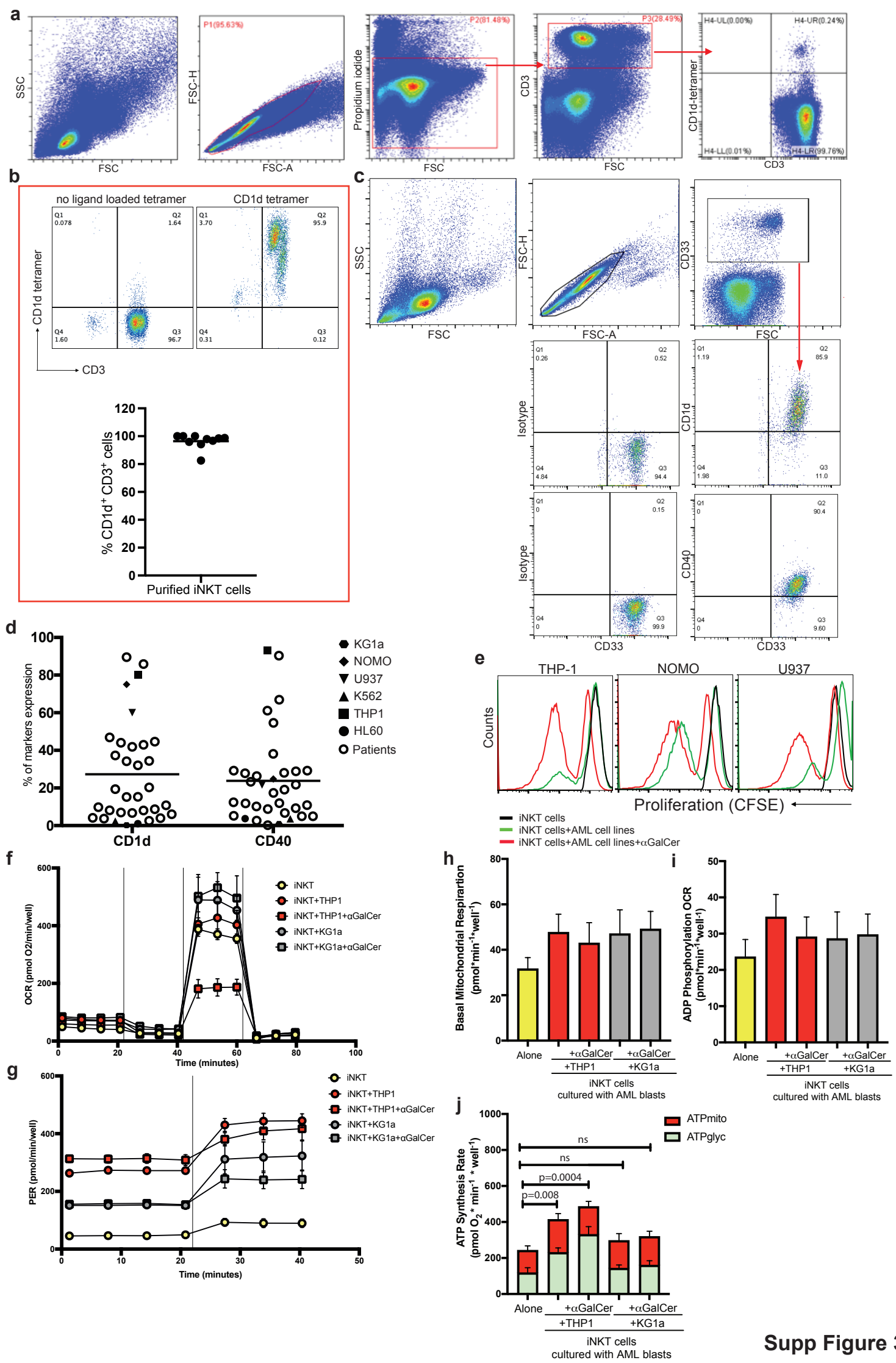

Supplement: Supplementary file 4 — Supplementary file4 (PDF 2721 KB) [file 262_2022_3268_MOESM4_ESM.pdf]

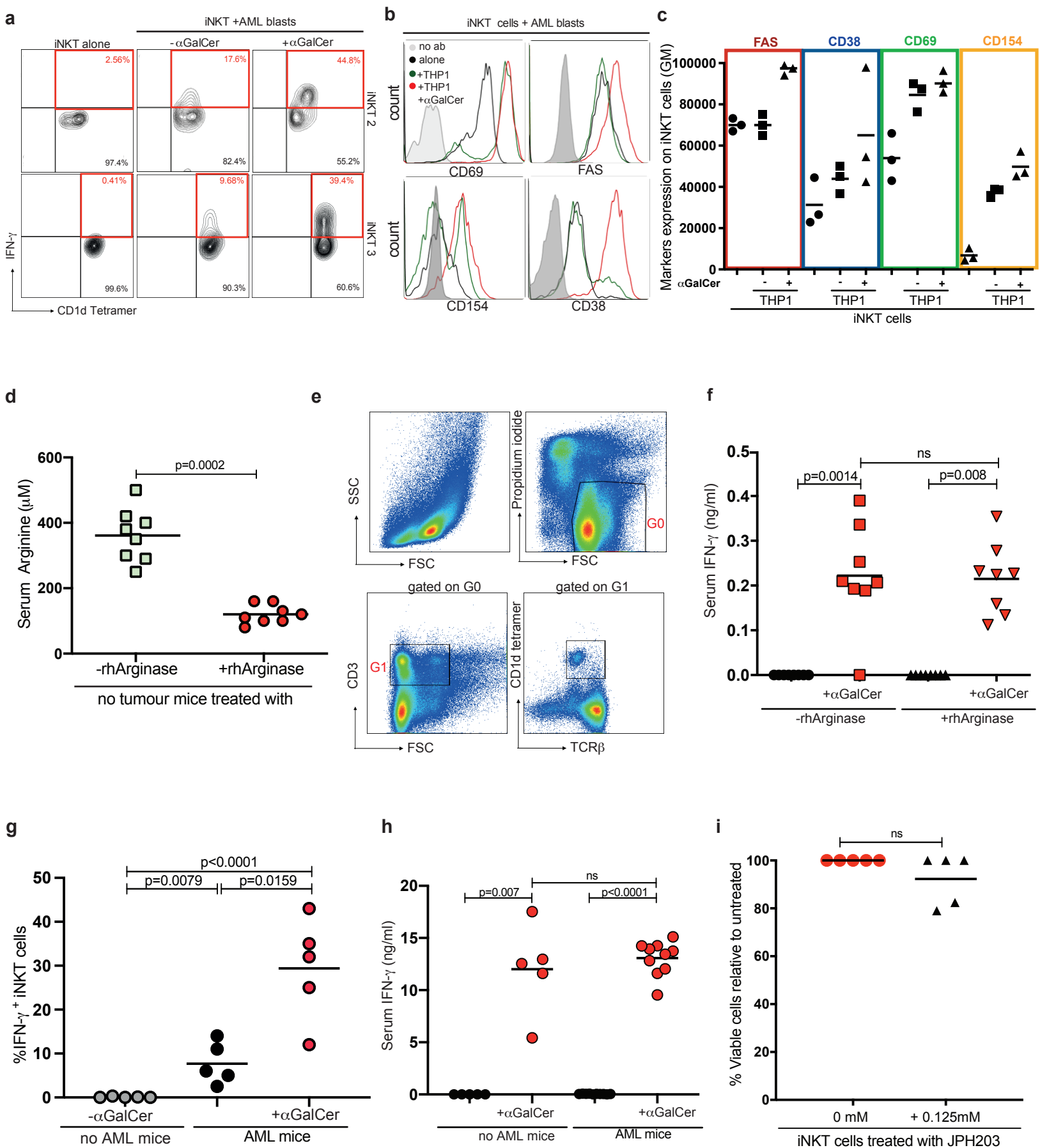

Supplement: Supplementary file 5 — Supplementary file5 (PDF 758 KB) [file 262_2022_3268_MOESM5_ESM.pdf]

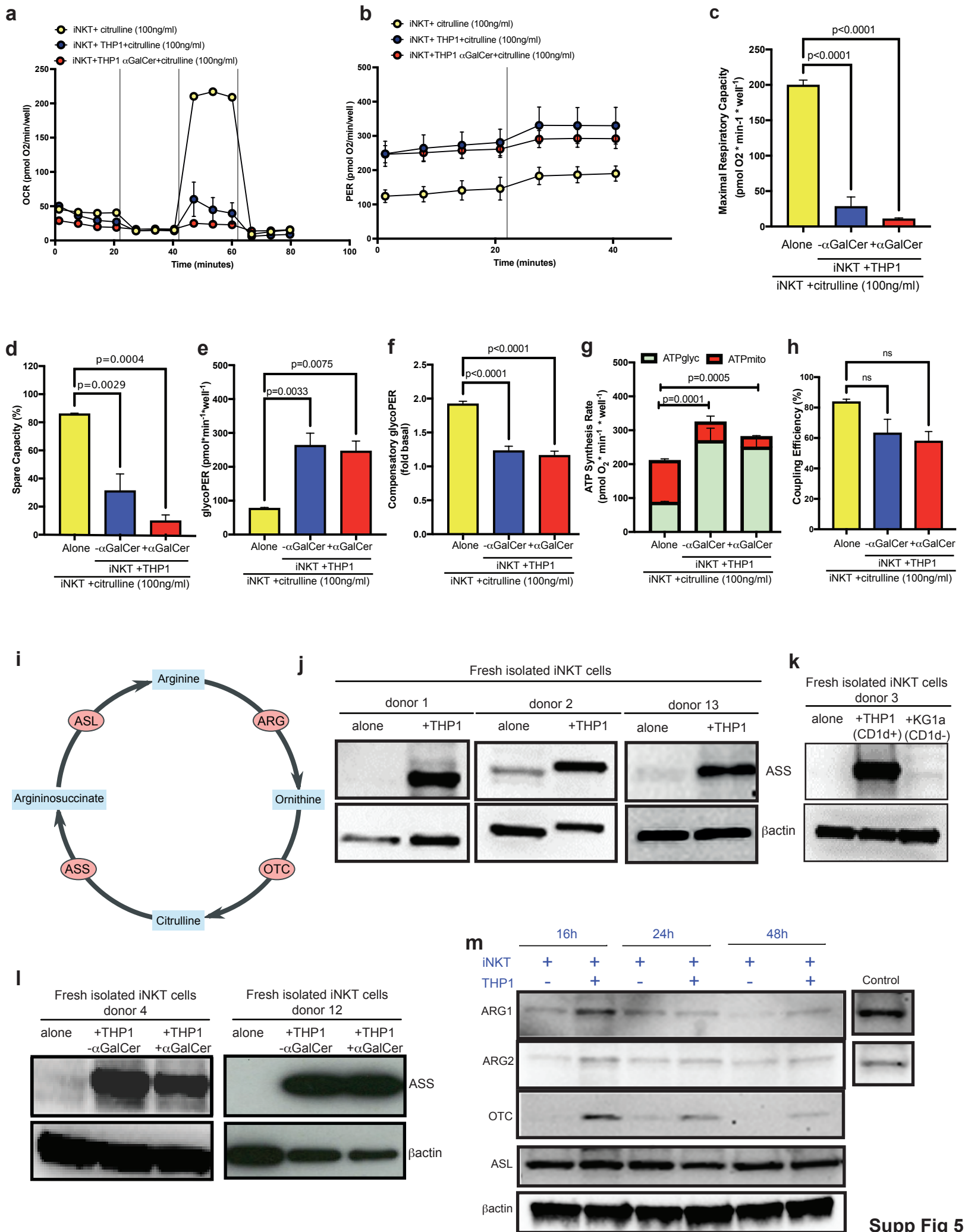

Supplement: Supplementary file 6 — Supplementary file6 (PDF 5980 KB) [file 262_2022_3268_MOESM6_ESM.pdf]

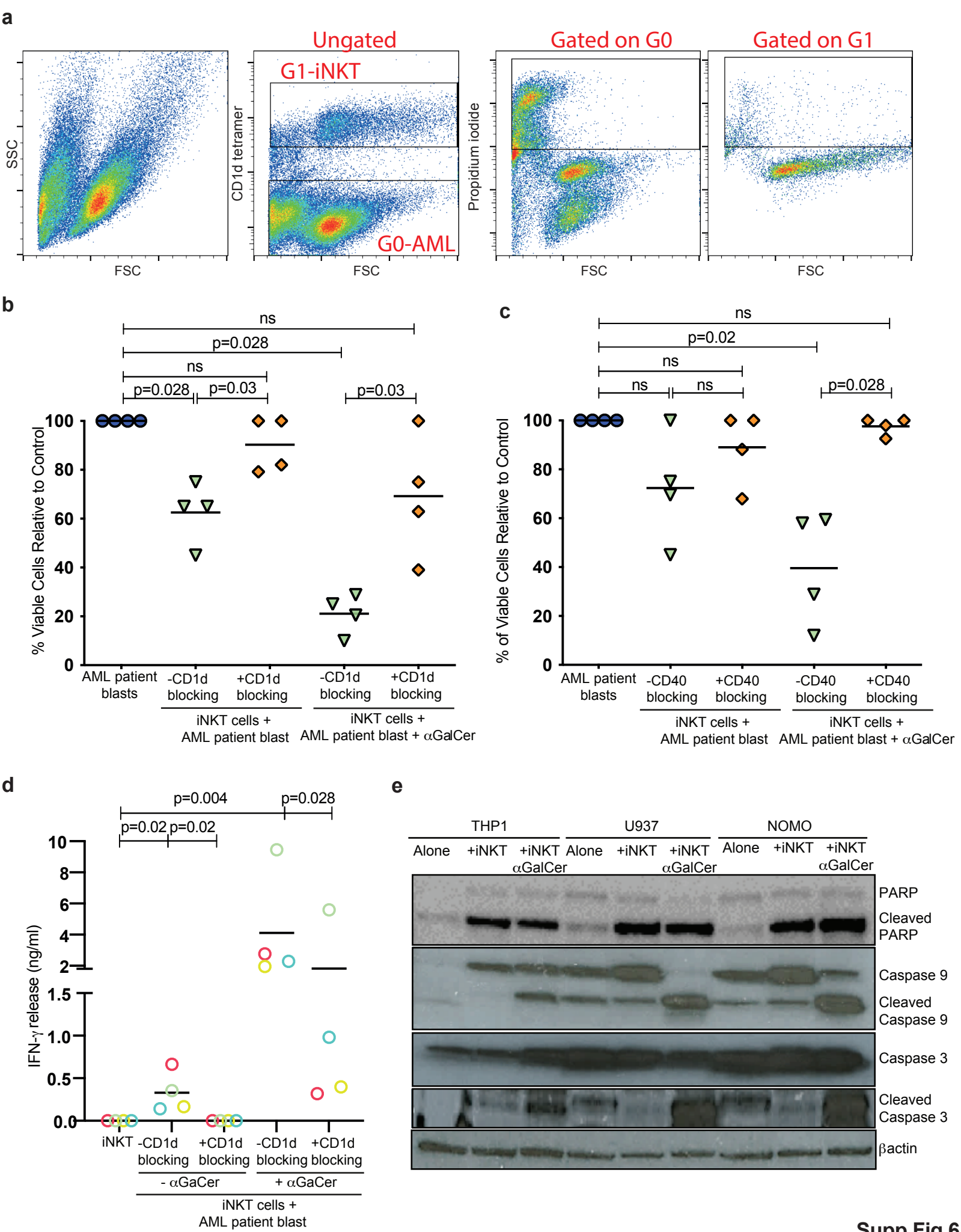

Supp Fig 6

Supplement: Supplementary file 7 — Supplementary file7 (PDF 780 KB) [file 262_2022_3268_MOESM7_ESM.pdf]

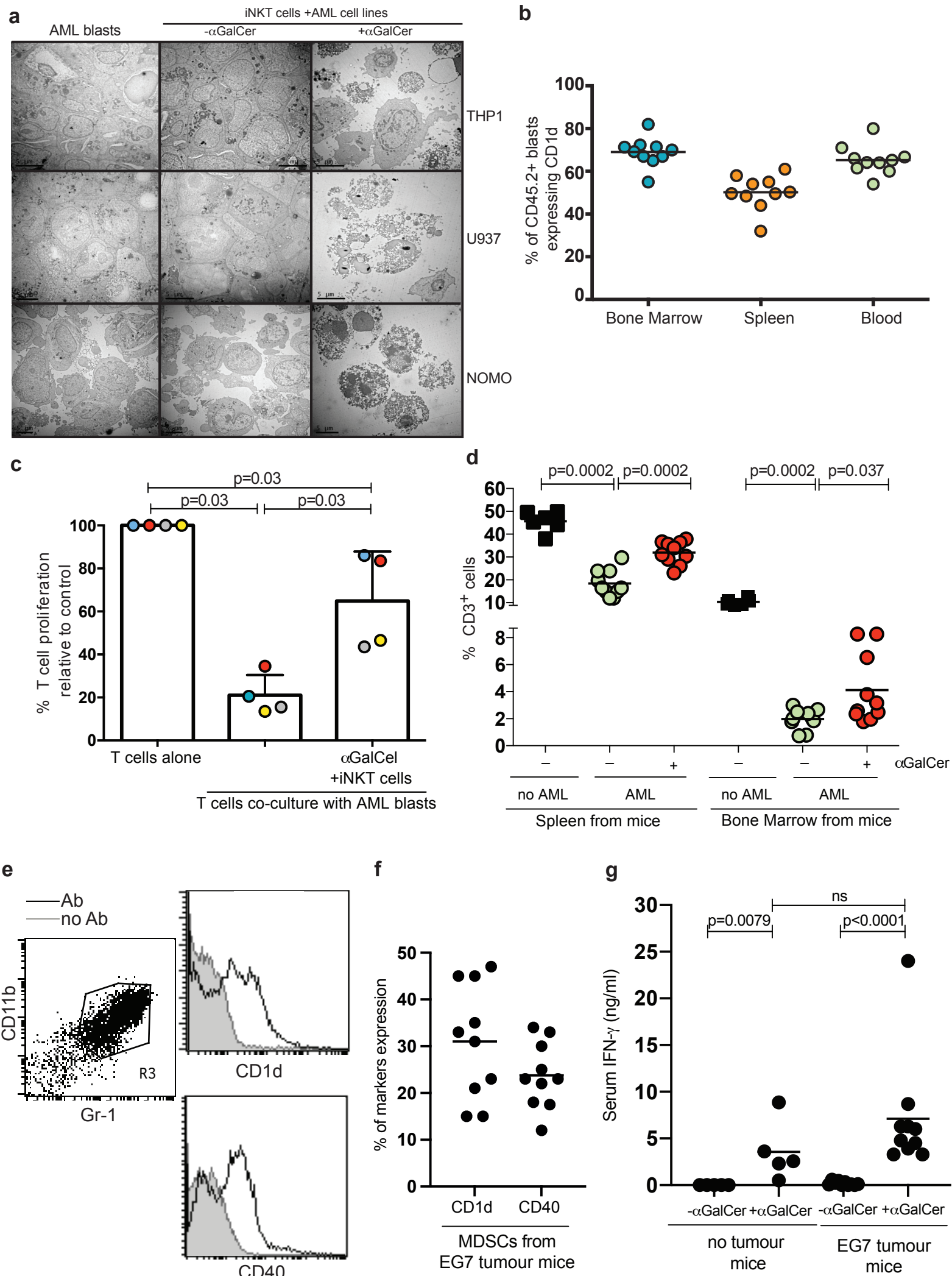

Supplement: Supplementary file 8 — Supplementary file8 (PDF 3595 KB) [file 262_2022_3268_MOESM8_ESM.pdf]

**a**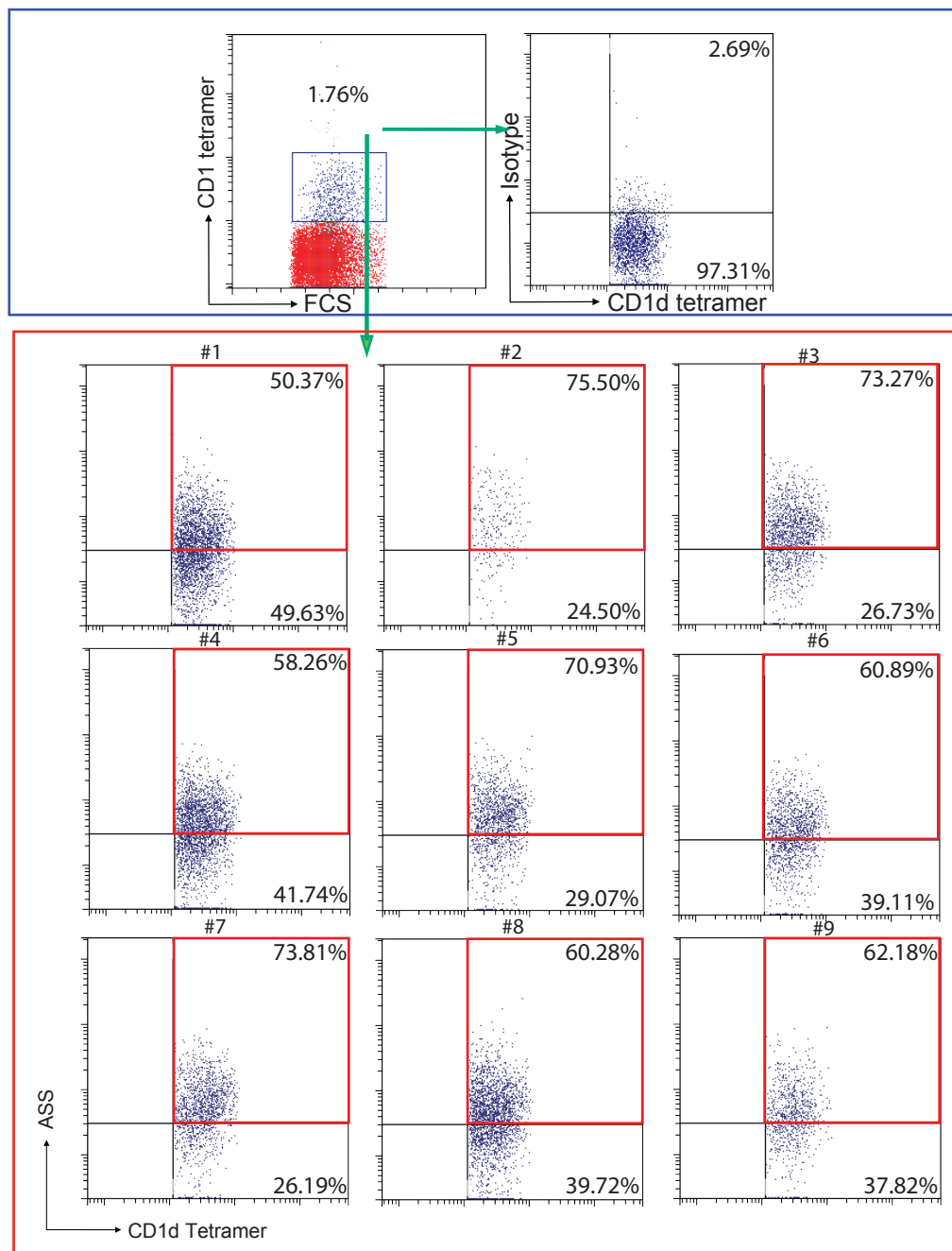**b**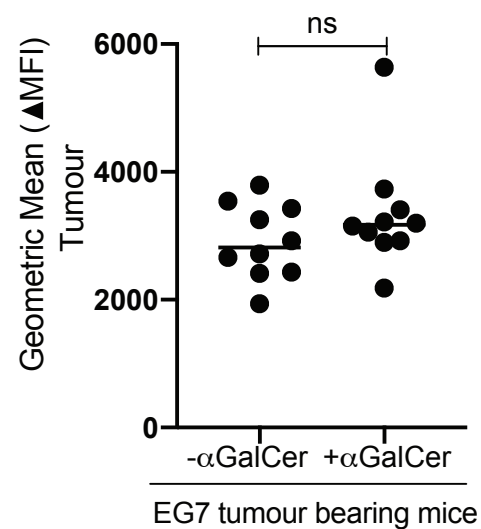**c**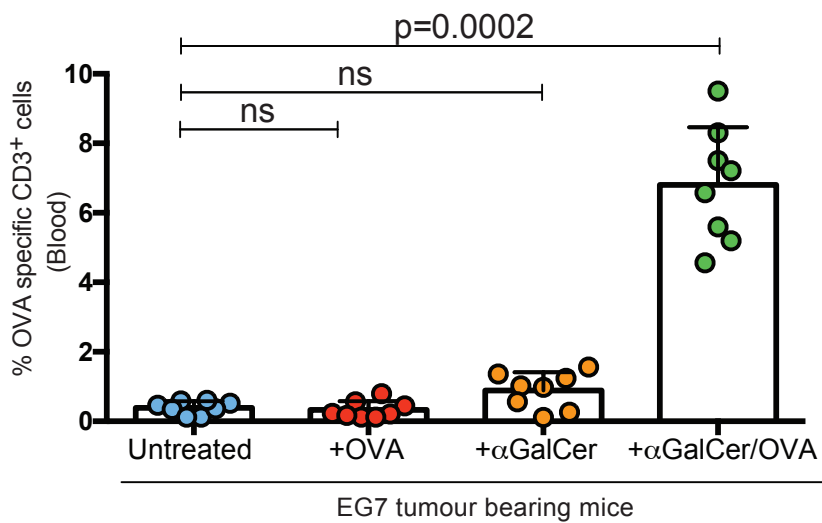**d**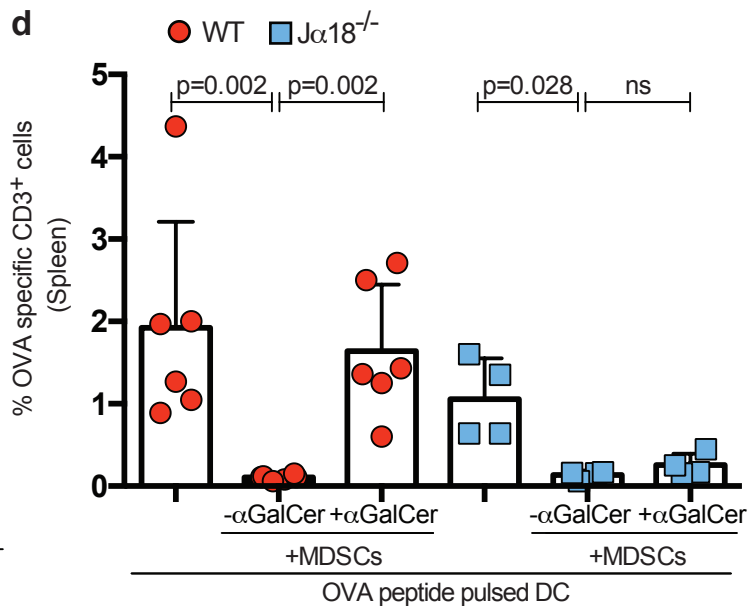

Supplement: Supplementary file 9 — Supplementary file9 (PDF 1364 KB) [file 262_2022_3268_MOESM9_ESM.pdf]
