# Supplementary material for: Invariant NKT cells metabolically adapt to the acute myeloid leukaemia environment
Source: Cancer Immunol Immunother. 2022 Aug 13;72(3):543–60. doi: 10.1007/s00262-022-03268-4 (PMC9947083; doi:10.1007/s00262-022-03268-4)
Supplement: Supplementary file 10 — Supplementary file10 (PDF 86 KB) [file 262_2022_3268_MOESM10_ESM.pdf]

Pathway Name	Match Status	P-value	Metabolite (metabolites in red are the metabolites which were statistically significant)
Pantothenate and CoA biosynthesis	5/27	0.002617	Dephospho-CoA; Coenzyme A; Apo-[acyl-carrier-protein]; Pantetheine 4'-phosphate; Acyl-carrier protein; Pantetheine; 4-Phosphopantothenoylcysteine; D-Pantothenoyl-L-cysteine; D-4'-Phosphopantothenate; L-Cysteine; Pantothenic acid; **Ureidopropionic acid**; **Dihydrouracil**; (R)-Pantoate; Beta-Alanine; Alpha-ketoisovaleric acid; L-Valine; **2,3-Dihydroxy-3-methylbutanoate**; **2-Acetolactate** ; **Pyruvic acid**; (R)-4-Dehydropantoate; (R)-3,3-Dimethylmalate; Pantothenol; L-Aspartic acid; Adenosine 3',5'-diphosphate; Uracil; 2-Dehydropantoate
Valine, leucine and isoleucine biosynthesis	5/27	0.002617	(R)-2-Methylmalate; **Pyruvic acid**; Acetyl-CoA; Citraconic acid; D-erythro-3-Methylmalate; L-Threonine; 3-Methyl-2-oxovaleric acid; **L-Leucine**; (R) 2,3-Dihydroxy-3-methylvalerate; 2-Isopropylmalic acid; **3-Isopropylmalate**; Alpha-ketoisovaleric acid; L-Valine; (R)-2,3-Dihydroxy-isovalerate; (S)-2-Aceto-2-hydroxybutanoic acid; (S)-2-Acetolactate; 2-(a-Hydroxyethyl)thiamine diphosphate; **L-Isoleucine**; **2-Ketobutyric acid**; 2-Isopropyl-3-oxosuccinate; L-Leucyl-tRNA; 4-Methyl-2-oxopentanoate; (R)-3-Hydroxy-3-methyl-2-oxopentanoate; Isopropylmaleate; L-Valyl-tRNA(Val); 3-Hydroxy-3-methyl-2-oxobutanoic acid; L-Isoleucyl-tRNA(Ile)
Arginine and proline metabolism	8/77	0.006607	L-Glutamic-gamma-semialdehyde; Pyrroline hydroxycarboxylic acid; **L-Glutamine**; Ammonia; Carbamoylphosphate; Ornithine; L-Aspartic acid; Citrulline; Argininosuccinic acid; **L-Arginine**; L-Glutamic acid; N-Acetyl-L-alanine; N-Acetyl-L-glutamyl 5-phosphate; N-Acetyl-L-glutamate 5-semialdehyde; N-Acetylornithine; L-Proline; Peptide; 4-Oxoproline; D-Proline; 1-Pyrroline-2-carboxylic acid; Hydroxyproline; L-4-Hydroxyglutamate semialdehyde; L-erythro-4-Hydroxyglutamate; D-4-Hydroxy-2-oxoglutarate; Nopaline; N-(o)-Hydroxyarginine; Guanidoacetic acid; **Creatine**; **N-Carbamoylsarcosine**; Phosphocreatine; **N-Methylhydantoin**; **Creatinine**; 5-Guanidino-2-oxopentanoate; 4-Guanidinobutanal; 4-Guanidinobutanoic acid; Gamma-Aminobutyric acid; 4-Guanidinobutanamide; Agmatine; N-Carbamoylputrescine; N2-Succinyl-L-arginine; N2-Succinyl-L-ornithine; N2-Succinyl-L-glutamic acid 5-semialdehyde; N-Succinyl-L-glutamate; L-Glutamic acid 5-phosphate; (S)-1-Pyrroline-5-carboxylate; Putrescine; Gamma-glutamyl-L-putrescine; gamma-Glutamyl-gamma-aminobutyraldehyde; 4-(Glutamylamino) butanoate; 4-Aminobutyraldehyde; S-Adenosylmethioninamine; S-Adenosylmethionine; Spermidine; N-Acetylputrescine; N4-Acetylaminobutanal; **4-Acetamidobutanoic acid**; Urea; Urea-1-carboxylate; cis-4-Hydroxy-D-proline; 1-Pyrroline-4-hydroxy-2-carboxylate; Fumaric acid; 5-Amino-2-oxopentanoic acid; 5-Aminopentanoic acid; **Pyruvic acid**; Glyoxylic acid; N2-(D-1-Carboxyethyl)-L-arginine; L-Arginine phosphate; Nitric oxide; Sarcosine; Spermine; Carbon dioxide; Homocarnosine; Phosphoguanidinoacetate; 2,5-Dioxopentanoate; Pyrrole-2-carboxylic acid; 2-Oxo-4-hydroxy-5-aminovalerate; Linatine
Lysine degradation	6/47	0.00683	D-Lysine; 2,5-Diaminohexanoate; **L-Lysine**; delta1-Piperideine-2-carboxylate; Saccharopine; 4-Trimethylammoniobutanoic acid; 4-Trimethylammoniobutanal; N6,N6,N6-Trimethyl-L-lysine; Protein N6,N6,N6-trimethyl-L-lysine; Protein N6-methyl-L-lysine; Protein lysine; 5-Hydroxylysine; **Protein N6,N6-dimethyl-L-lysine**; Crotonoyl-CoA; (S)-3-Hydroxybutanoyl-CoA; **Pipecolic acid**; N6-Acetyl-L-lysine; L-2-Aminoadipate 6-semialdehyde; Aminoadipic acid; Oxoadipic acid; Acetoacetyl-CoA; Glutaryl-CoA; **Glutaric acid**; Glutarate semialdehyde; 5-Aminopentanamide; 5-Acetamidopentanoate; 2-Keto-6-acetamidocaproate; (3S)-3,6-Diaminohexanoate; (3S,5S)-3,5-Diaminohexanoate; N6-Acetyl-N6-hydroxy-L-lysine; N6-Hydroxy-L-lysine; 3-Hydroxy-N6,N6,N6-trimethyl-L-lysine; S-Glutaryldihydrolipoamide; N2-(D-1-Carboxyethyl)-L-lysine; **2-Keto-6-aminocaproate**; 2-Amino-5-oxohexanoate; Carnitine; 5-(D-Galactosyloxy)-L-lysine-procollagen; 5-Phosphonooxy-L-lysine; (S)-2,3,4,5-Tetrahydropyridine-2-carboxylate; Acetyl-CoA; 5-Aminopentanoic acid; **(S)-5-Amino-3-oxohexanoate**; Aerobactin; Glycine; Piperideine
Glycine, serine and threonine metabolism	6/48	0.007578	**Betaine aldehyde**; L-Serine; Ectoine; Choline; N-gamma-Acetyldiaminobutyrate; L-2,4-Diaminobutanoate; L-Aspartate-semialdehyde; 3-Phospho-D-glycerate; Glyceric acid; Betaine; Guanidoacetic acid; Dimethylglycine; L-Cystathionine; Glycine; L-Aspartic acid; Phosphoserine; Sarcosine; 5,10-Methylene-THF; L-Threonine; O-Phosphohomoserine; L-Aspartyl-4-phosphate; **L-Homoserine**; Lipoylprotein; D-Serine; Aminoacetone; Pyruvaldehyde; Tetrahydrofolic acid; S-Aminomethyldihydrolipoylprotein; D-Lombricine; Dihydrolipoylprotein; **Creatine**; 5-Hydroxyectoine; Hydroxypyruvic acid; Phosphohydroxypyruvic acid; L-Cysteine; **L-Allothreonine**; **2-Ketobutyric acid**; Glyoxylic acid; L-2-Amino-3-oxobutanoic acid; **Pyruvic acid**; Carbon dioxide; 5-Aminolevulinic acid; Hydroxyacetone; (R)-1-Aminopropan-2-ol; Ammonia; N-Phospho-D-lombricine; PS(16:0/16:0); L-Tryptophan
Alanine, aspartate and glutamate metabolism	4/24	0.010528	N-Acetyl-L-aspartic acid; 2-Oxosuccinamate; L-Aspartic acid; **L-Asparagine**; D-Aspartic acid; Argininosuccinic acid; Adenylsuccinic acid; L-Alanine; **Pyruvic acid**; Ureidosuccinic acid; **Succinic acid semialdehyde**; Oxoglutaric acid; **L-Glutamine**; L-Glutamic acid; Gamma-Aminobutyric acid; Ammonia; 2-Keto-glutaramic acid; (S)-1-Pyrroline-5-carboxylate; Oxalacetic acid; Fumaric acid; Succinic acid; Carbamoylphosphate; Glucosamine 6-phosphate; 5-Phosphoribosylamine
beta-Alanine metabolism	4/28	0.018129	Beta-Alanyl-CoA; Acrylyl-CoA; 3-Hydroxypropionyl-CoA; Hydroxypropionic acid; Malonic acid; Malonyl-CoA; **Malonic semialdehyde**; Beta-Alanine; beta-Alanyl-L-lysine; L-Aspartic acid; N-Acetyl-beta-alanine; Spermine; Spermidine; 4-Aminobutyraldehyde; **1,3-Diaminopropane**; 3-Aminopropionaldehyde; **Ureidopropionic acid**; **Dihydrouracil**; Carnosine; Anserine; beta-Alanyl-L-arginine; Propionyl-CoA; Acetyl-CoA; Propynoic acid; Pantothenic acid; Gamma-Aminobutyric acid; Uracil; L-Histidine
Aminoacyl-tRNA biosynthesis	7/75	0.019128	tRNA(Asn); **L-Asparagine**; tRNA(His); L-Histidine; tRNA(Phe); L-Phenylalanine; **L-Arginine**; tRNA(Arg); tRNA(Gln); **L-Glutamine**; tRNA(Cys); L-Cysteine; tRNA(Gly); Glycine; tRNA(Asp); L-Aspartic acid; tRNA(Ser); L-Serine; L-Methionine; tRNA(Met); L-Valine; tRNA(Val); tRNA(Ala); L-Alanine; tRNA(Lys); **L-Lysine**; tRNA(Ile); **L-Isoleucine**; tRNA(Leu); **L-Leucine**; L-Threonine; tRNA(Thr); tRNA(Trp); L-Tryptophan; L-Methionyl-tRNA; N10-Formyl-THF; **L-Tyrosine**; tRNA(Tyr); L-Proline; tRNA(Pro); tRNA(Glu); L-Glutamic acid; Glutaminyl-tRNA; L-Asparaginyl-tRNA(Asn); O-Phosphoseryl-tRNA(Cys); Phosphoserine; tRNA(Sec); L-Seryl-tRNA(Sec); O-Phosphoseryl-tRNA(Sec); L-Pyrrolysine; tRNA(Pyl); L-Histidyl-tRNA(His); L-Phenylalanyl-tRNA(Phe); L-Arginyl-tRNA(Arg); L-Cysteinyl-tRNA(Cys); Glycyl-tRNA(Gly); L-Aspartyl-tRNA(Asp); L-Seryl-tRNA(Ser); L-Valyl-tRNA(Val); L-Alanyl-tRNA; L-Lysyl-tRNA; L-Isoleucyl-tRNA(Ile); L-Leucyl-tRNA; L-Threonyl-tRNA(Thr); L-Tryptophanyl-tRNA(Trp); Tetrahydrofolic acid; N-Formylmethionyl-tRNA; L-Tyrosyl-tRNA(Tyr); L-Prolyl-tRNA(Pro); L-Glutamyl-tRNA(Glu); L-Glutamyl-tRNA(Gln); L-Aspartyl-tRNA(Asn); L-Selenocysteinyl-tRNA(Sec); L-Pyrrolysyl-tRNA(Pyl); L-Lysyl-tRNA(Pyl)
Pyrimidine metabolism	6/60	0.021697	Uridine 5'-diphosphate; Thioredoxin; 3-Oxo-3-ureidopropanoate; Uridine 5'-monophosphate; dCTP; dUMP; **L-Glutamine**; Carbamoylphosphate; 4,5-Dihydroorotic acid; **Orotidylic acid**; RNA; Uridine triphosphate; Cytidine triphosphate; Uridine; **Dihydrouracil**; **Ureidopropionic acid**; CDP; Cytidine monophosphate; Cytidine; Cytosine; Uracil; Barbiturate; Thioredoxin disulfide; dCDP; dCMP; Deoxycytidine; Deoxyuridine triphosphate; dUDP; Deoxyuridine; Thymidine 5'-triphosphate; dTDP; 5-Thymidylic acid; Thymidine; 5-Methylcytosine; Thymine; **Dihydrothymine**; 5-Methylbarbiturate; **Ureidoisobutyric acid**; Pseudouridine; Uridine diphosphate glucose; 3'-UMP; 2',3'-Cyclic UMP; 3'-CMP; 2',3'-Cyclic CMP; Trimetaphosphate; P1,P4-Bis(5'-uridyl) tetraphosphate; 5-Hydroxymethyldeoxycytidylate; 2'-Deoxy-5-hydroxymethylcytidine-5'-diphosphate; Malonic acid; Urea; Ureidosuccinic acid; Orotic acid; Phosphoribosyl pyrophosphate; Beta-Alanine; DNA; Deoxyribose 1-phosphate; Methylmalonic acid; 3-Aminoisobutanoic acid; Pseudouridine 5'-phosphate; 2'-Deoxy-5-hydroxymethylcytidine-5'-triphosphate
Phenylalanine metabolism	5/45	0.023493	4-Hydroxy-2-oxopentanoate; L-Phenylalanine; Phenylacetaldehyde; Phenylacetic acid; Phenylacetyl-CoA; 2-Hydroxy-2,4-pentadienoate; 2-Hydroxy-6-oxonona-2,4-diene-1,9-dioate; 2-Hydroxy-6-ketononatrienedioate; 3-(2,3-Dihydroxyphenyl)propanoate; trans-2,3-Dihydroxycinnamate; m-Coumaric acid; Phenylethylamine; Phenylpyruvic acid; Phenyllactate; D-Phenylalanine; Phenylethyl alcohol; 2-Phenylacetamide; **trans-Cinnamic acid**; cis-3-(3-Carboxyethenyl)-3,5-cyclohexadiene-1,2-diol; 3-(2-Hydroxyphenyl)propanoate; 3-(3-Hydroxyphenyl)propanoic acid; cis-3-(Carboxy-ethyl)-3,5-cyclo-hexadiene-1,2-diol; Hydrocinnamic acid; Benzoic acid; **Hippuric acid**; trans-2-Hydroxycinnamate; 4-Hydroxycinnamic acid; Phenylglyoxylic acid; Phenylglyoxylyl-CoA; **Pyruvic acid**; Acetaldehyde; **Alpha-N-Phenylacetyl-L-glutamine**; Phenylacetylglycine; Succinic acid; Fumaric acid; Ortho-Hydroxyphenylacetic acid; Enol-phenylpyruvate; N-Acetyl-D-phenylalanine; N-Acetyl-L-phenylalanine; Benzoyl-CoA; 4-Hydroxybenzoic acid; p-Hydroxyphenylacetic acid; Salicylic acid; **L-Tyrosine**; 3-Hydroxyphenylacetic acid
Cysteine and methionine metabolism	5/56	0.053576	O-Succinyl-L-homoserine; 1-Aminocyclopropane-1-carboxylate; S-Adenosylmethionine; 2-Oxo-4-methylthiobutanoic acid; 2-Hydroxy-3-keto-5-methylthiopentenyl-1-phosphate; 1,2-Dihydroxy-3-keto-5-methylthiopentene; 2,3-Diketo-5-methylthiopentyl-1-phosphate; 5-Methylthioribulose 1-phosphate; 5-Methylthioribose 1-phosphate; 5-Methylthioribose; 5'-Methylthioadenosine; S-Adenosylmethioninamine; L-Methionine S-oxide; DL-Homocystine; L-Cystathionine; N-Formyl-L-methionine; L-Homocysteine; L-Serine; L-Methionine; S-Adenosylhomocysteine; S-Ribosyl-L-homocysteine; O-Acetylserine; Hydrogen sulfide; S-Glutathionyl-L-cysteine; Sulfate; Glutathione; L-Cysteine; 2-Aminoacrylic acid; Phosphoserine; Cysteic acid; 3-Sulfopyruvic acid; 3-Sulfolactate; **L-Cystine**; 3-Sulfinoalanine; 3-Sulfinylpyruvic acid; D-Cysteine; Sulfite; 3-Mercaptopyruvic acid; **L-Homoserine**; O-Acetyl-L-homoserine; L-Aspartyl-4-phosphate; L-Aspartic acid; Ethylene; **3-Methylthiopropionic acid**; **2-Ketobutyric acid**; Methanethiol; Cysteine-S-sulfate; **Pyruvic acid**; Hydrogen sulfite; Thiocysteine; Thiosulfate; 3-Mercaptolactic acid; L-Aspartate-semialdehyde; Aminoacyl-L-methionine; L-Alanine; Sulfur dioxide
Valine, leucine and isoleucine degradation	4/40	0.057939	Enzyme N6-(lipoyl)lysine; 2-Methyl-1-hydroxybutyl-ThPP; Enzyme N6-(dihydrolipoyl)lysine; 2-Methyl-1-hydroxypropyl-ThPP ; 3-Methyl-1-hydroxybutyl-ThPP; Acetyl-CoA; **Beta-Leucine**; **L-Leucine**; Acetoacetyl-CoA; **Acetoacetic acid**; 3-Hydroxy-3-methylglutaryl-CoA; 3-Methylcrotonyl-CoA; 3-Hydroxyisovaleryl-CoA; Isovaleryl-CoA; Thiamine pyrophosphate; 3-Methyl-2-oxovaleric acid; L-Valine; 2-Methylacetoacetyl-CoA; (S)-3-Hydroxyisobutyrate; Tiglyl-CoA; Butyryl-CoA; S-(2-Methylbutanoyl)-dihydrolipoamide; Alpha-ketoisovaleric acid; **L-Isoleucine**; R-Methylmalonyl-CoA; Methylmalonyl-CoA; Propionyl-CoA; (S)-Methylmalonic acid semialdehyde; (S)-b-aminoisobutyric acid; 2-Methyl-3-hydroxybutyryl-CoA; (S)-3-Hydroxyisobutyryl-CoA; Methacrylyl-CoA; (S)-2-Methylbutanoyl-CoA; S-(2-Methylpropionyl)-dihydrolipoamide-E; 4-Methyl-2-oxopentanoate; S-(3-Methylbutanoyl)-dihydrolipoamide-E; beta-Ketoisocaproate; 3-Methylglutaconyl-CoA; Succinyl-CoA; Methylmalonic acid
Butanoate metabolism	4/40	0.057939	3-Butyn-1-ol; 3-Butyn-1-al; 3-Butynoate; (R)-3-Hydroxybutyric acid; (R)-3-((R)-3-Hydroxybutanoyloxy)butanoate; **Acetoacetic acid**; 3-Hydroxy-3-methylglutaryl-CoA; Acetyl-CoA; Acetoacetyl-CoA; (S)-3-Hydroxybutanoyl-CoA; 3-Hydroxybutyryl-CoA; Poly-beta-hydroxybutyrate; Crotonoyl-CoA; Vinylacetyl-CoA; 4-Hydroxybutyric acid; Gamma-Aminobutyric acid; L-Glutamic acid; **Pyruvic acid**; Butanoyl-CoA; Butanal; **Succinic acid semialdehyde**; Butyric acid; (R)-Malate; Maleic acid; Succinic acid; Thiamine pyrophosphate; 2-(a-Hydroxyethyl)thiamine diphosphate; **2-Acetolactate** ; (S)-Acetoin; (R)-Acetoin; 2-Hydroxyglutaryl-CoA; 2-Hydroxyglutarate; Glutaconyl-1-CoA; Oxoglutaric acid; Butanoylphosphate; 1-Butanol; Fumaric acid; (R,R)-Butane-2,3-diol; (S,S)-Butane-2,3-diol; Diacetyl
